# Red-emitting chemosensor detects superoxide anion in models of myocardial hypertrophy

**DOI:** 10.1039/d6ra00466k

**Published:** 2026-09-23

**Authors:** Fengting Guo, Yonghui Liu, Qiu Chen, Yasheng Deng

**Affiliations:** a Guangzhou University of Chinese Medicine Guangzhou China; b Guangxi University of Chinese Medicine Nanning China; c Hospital of Chengdu University of Traditional Chinese Medicine Chengdu China; d Fangchenggang Hospital of Traditional Chinese Medicine Fangchenggang China dengyasheng@stu.cdutcm.edu.cn

## Abstract

Cardiac hypertrophy is a compensatory pathological alteration of the heart in response to prolonged stress, for which suitable diagnostic methods are lacking. Superoxide anion (O_2_˙^−^) levels are considered to be elevated during the progression of this disease. A red-emitting fluorescent chemosensor DO was rationally designed and synthesized. It enabled sensitive and accurate detection of O_2_˙^−^ in solution, with a low limit of detection (LOD) of 93 nM. Chemosensor DO detected the increase in O_2_˙^−^ concentration induced by angiotensin II (Ang II) *in vitro* and showed that losartan antagonizes Ang II-induced O_2_˙^−^ production. Chemosensor DO was applied to detect elevated superoxide levels in heart tissue sections from hypertrophic mice. Therefore, this probe provides a new perspective for understanding the pathogenesis of cardiac hypertrophy.

## Abstract 2

An endoplasmic reticulum-targeted dicyanoisophorone-based fluorescent probe DO was developed for superoxide anion detection, enabling real-time imaging of oxidative stress in Ang II-induced myocardial hypertrophy in living mice.

## Graphical abstract

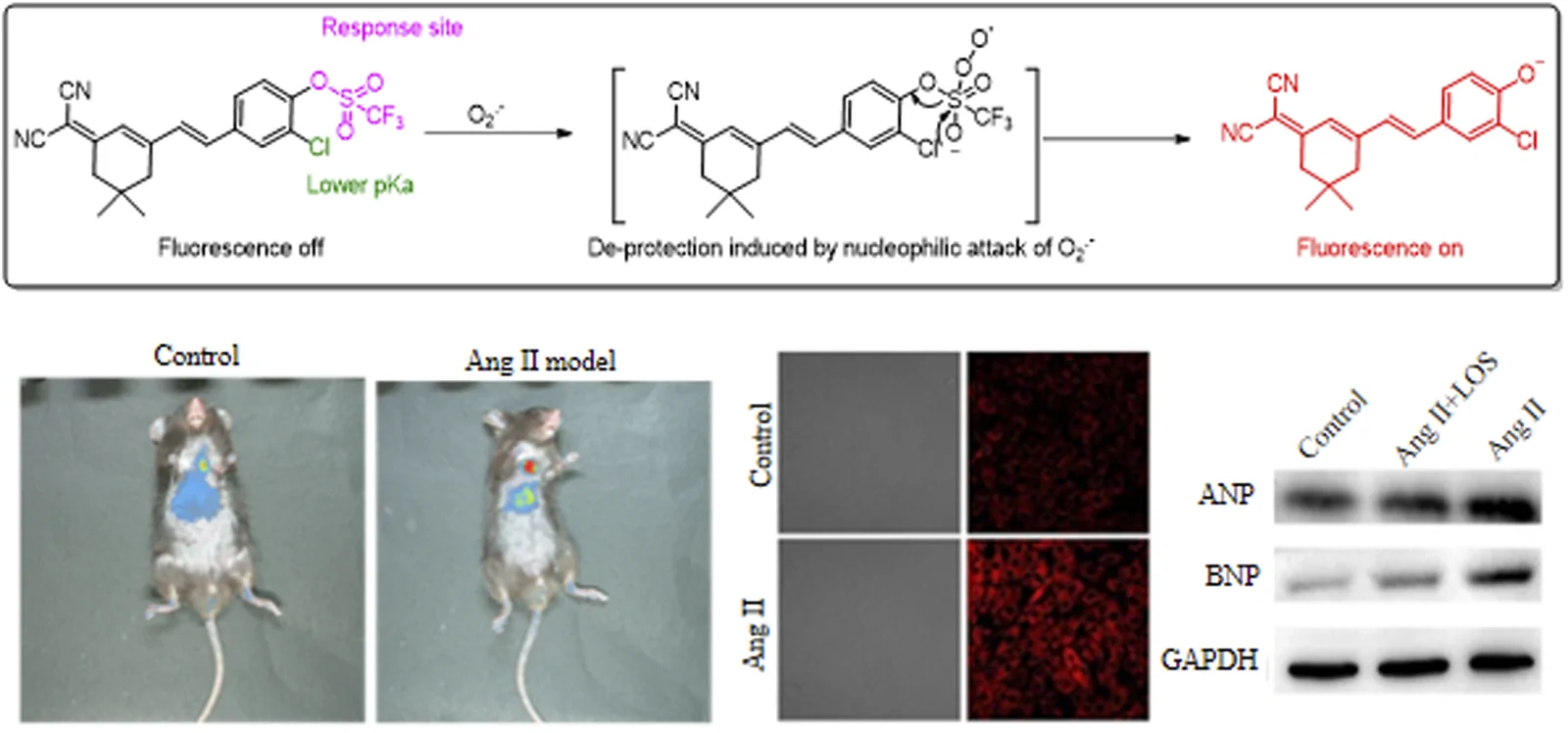

## Introduction

Myocardial hypertrophy is a pathological condition characterized by an increase in cardiomyocyte volume and thickening of the myocardial wall.^1,2^ Its core etiology involves the myocardium enduring prolonged abnormal mechanical load or stimulation by neurohumoral factors, leading to myocardial remodeling.^3^

The diagnosis of myocardial hypertrophy primarily relies on imaging, electrocardiograms (ECGs), and blood biomarkers.^4–6^ Cardiac imaging can clearly visualize myocardial thickness and contractile amplitude, serving as the most persuasive diagnostic method available. However, this approach lacks sufficient sensitivity for detecting mild hypertrophy and is associated with high equipment costs. ECG offers the advantages of convenience and low cost in diagnosing patients with myocardial hypertrophy,^7,8^ yet it suffers from low specificity.^9,10^ Blood tests aid in disease diagnosis by measuring the concentration of myocardial injury markers (such as troponin and creatine kinase-MB) and neurohumoral factors (such as atrial natriuretic peptide (ANP) and brain natriuretic peptide (BNP)). However, there are no specific biomarkers directly targeting the pathological changes seen in myocardial hypertrophy.^11,12^ Therefore, there is an urgent need to identify specific biomarkers for myocardial hypertrophy to facilitate early disease diagnosis and therapeutic efficacy evaluation.

Reactive oxygen species (ROS) levels have been found to be significantly elevated at the early stages of myocardial hypertrophy progression. For example, in the aortic constriction model in rats, ROS levels within myocardial tissue increase significantly even before the onset of hypertrophy, and there is a dose-dependent positive correlation between ROS levels and the degree of myocardial hypertrophy.^13–15^ A similar phenomenon has been observed in the Ang II infusion model in mice.^1,16^ This could be because increased mechanical load and activation of neurohumoral factors induce excessive ROS production, which, in turn, activates mitogen-activated protein kinase (MAPK) and phosphatidylinositol 3-kinase/protein kinase B (PI3K/Akt) pathways. This leads to the nuclear translocation of transcription factors (*e.g.*, nuclear factor of activated T cells (NFAT) and GATA binding protein 4 (GATA4)), upregulates the expression of hypertrophy-associated genes (including ANP, BNP, and β-myosin heavy chain (β-MHC)) and, ultimately, drives cardiomyocyte hypertrophy.^16–19^ Therefore, ROS can serve as a promising biomarker for myocardial hypertrophy.

ROS is a collective term for oxygen-containing molecules or ions with high reactivity. The primary types include O_2_˙^−^, hydrogen peroxide (H_2_O_2_), hydroxyl radical (˙OH), and peroxynitrite (ONOO^−^), among others.^20^ O_2_˙^−^ is the first ROS generated in the body. It is produced by the single-electron reduction of oxygen molecules, catalyzed by the mitochondrial respiratory chain, NADPH oxidase, xanthine oxidase, or directly excited by ultraviolet light and ionizing radiation. O_2_˙^−^ can react further to generate other ROS.^21^ In other words, it can, to some extent, represent the concentration of total ROS. Therefore, detecting O_2_˙^−^ in organisms is important for elucidating the biological functions of ROS.

Methods for the *in vivo* detection of O_2_˙^−^ primarily include electron spin resonance (ESR), enzymatic assays, and electrochemical methods.^22–24^ These methods can enable the measurement of O_2_˙^−^ concentrations within organisms. However, most of these methods are based on the analysis of *ex vivo* samples, which are highly destructive to biological specimens and lack spatiotemporal resolution. Therefore, there is a need for a detection method with high biocompatibility that enables *in vivo* imaging to bridge the gap between detection capabilities and biological safety. The core advantages of this sensing strategy lie in its high spatiotemporal resolution, biocompatibility for *in vivo* applications, operational convenience, and visualization capabilities, making it perfectly suited for the *in situ* detection of biological samples (such as cells, tissues, and live animals).^25–27^

In the present study, to monitor the dynamics of O_2_˙^−^ in myocardial hypertrophy, we designed a novel fluorescent chemosensor. The latter responded specifically to O_2_˙^−^ in aqueous solution, cardiomyocytes, and myocardial tissues, emitting a red fluorescence signal as the readout. With its assistance, we identified O_2_˙^−^ to be a key factor in myocardial hypertrophy that can act as an auxiliary indicator to evaluate hypertrophic oxidative stress.

## Materials and methods

### General information and reagents

All chemicals, organic solvents, and bioanalytes were purchased from commercial suppliers and used as received, unless specified otherwise. Detailed descriptions regarding instrumentation, preparation of test solutions, spectral analysis protocols, cell culture procedures, and fluorescence imaging are provided in the SI.

### Synthesis of fluorescent chemosensor DO

Compound 1 (162 mg) was dissolved in dry dichloromethane (DCM, 5 mL) under a nitrogen atmosphere and cooled to −30 °C using an ethanol–ice bath. Triethylamine (TEA, 150 mg) was added dropwise, causing the solution to change from red to dark red. After 5 min, trifluoromethanesulfonic anhydride (564 mg) was slowly injected into the reaction mixture. The solution gradually turned pale yellow within 30 min. The reaction progress was monitored by thin-layer chromatography (TLC). After 1 h, the reaction was quenched by pouring the mixture into ice water (10 mL). The aqueous phase was extracted with dichloromethane (20 mL × 3). The combined organic layers were dried over anhydrous sodium sulfate (Na_2_SO_4_) and concentrated *in vacuo*. The crude product was purified by column chromatography (CC) ((DCM : petroleum ether (PE) = 1 : 1 v/v) to afford the yellow solid DO (197 mg, 86% yield). ^1^H nuclear magnetic resonance (^1^H NMR, 400 MHz, CDCl_3_) *δ* 7.65 (d, *J* = 2.0 Hz, 1H), 7.46 (dd, *J* = 8.6, 2.0 Hz, 1H), 7.38 (d, *J* = 8.6 Hz, 1H), 7.03–6.92 (m, 2H), 6.90 (s, 1H), 2.62 (s, 2H), 2.45 (s, 2H), 1.09 (s, 6H). ^13^C nuclear magnetic resonance (^13^C NMR, 100 MHz, CDCl3) *δ* 168.81, 152.18, 145.65, 137.17, 132.94, 132.12, 129.67, 128.05, 126.87, 125.19, 123.54, 113.08, 112.33, 80.50, 42.93, 39.16, 32.07, 28.01.

### *In vitro* assay of the fluorescent chemosensor

Fluorescent chemosensor DO (10 µM) was treated with various concentrations of O_2_˙^−^ (0–100 µM) in dimethyl sulfoxide (DMSO). After a 1-min reaction, the mixture was diluted to 1 mL with phosphate-buffered saline (PBS, 10 mM, pH 7.4) and incubated for an additional 10 min prior to analyses. The final assay solution contained 30% (v/v) DMSO, while 0.1% DMSO was used in all cell and tissue tests. Fluorescence measurements were performed at *λ*_ex_ = 500 nm, *λ*_em_ = 698 nm.

### Preparation of ROS and other analytes

O_2_˙^−^ was generated by sonicating potassium superoxide (KO_2_) in dry DMSO. The concentration of O_2_˙^−^ was determined spectrophotometrically from its absorbance at 250 nm (*ε* = 2682 M^−1^ cm^−1^). ONOO^−^ was synthesized by adding 0.6 M sodium nitrite (NaNO_2_), 0.6 M hydrochloric acid (HCl), and 0.7 M H_2_O_2_ simultaneously to a 3 M sodium hydroxide (NaOH) solution maintained at 0 °C. The concentration of the resulting peroxynitrite was determined using an extinction coefficient of 1670 M^−1^ cm^−1^ at 302 nm in 0.1 M NaOH aqueous solution. H_2_O_2_ solutions were prepared by appropriate dilution of a 30% aqueous stock solution. The concentration was determined by measuring the absorbance at 240 nm (*ε* = 43.6 M^−1^ cm^−1^). ˙OH radicals were generated *via* the Fenton reaction using iron(ii) chloride (FeCl_2_, 1.0 mM) and H_2_O_2_ (200 µM) in deionized (DI) water. NO was obtained from a stock solution of sodium nitroprusside. All other analytes were commercially available and used as received after dissolving them in deionized water.

### Fluorescence imaging experiments with cells

Living RAW264.7 and H9c2 cells were harvested and reseeded onto 15-mm glass-bottom dishes 24 h prior to confocal imaging. The latter was performed using a Zeiss 880 NLO microscope equipped with ZEN 2 lite software. Fluorescence images of the fluorescent chemosensor were acquired through the red channel using an excitation wavelength of 488 nm and an emission detection range of 650–750 nm. For quantitative analyses, the average fluorescence intensity per image was calculated by selecting defined regions of interest. Each experiment was independently repeated five times with consistent results. Data are presented as the mean ± standard deviation (S.D).

### Animal experiments

Male C57BL/6 mice (4–6 weeks) were purchased from Hunan Slack Jingda Laboratory Animal Co., Ltd (Changsha, China), with the experimental animal production license number SCXK (Xiang) 2024-0009. Mice were randomly divided into two groups: the cardiac hypertrophy model group and the control group. Mice in the model group received intraperitoneal injections of angiotensin II (Ang II) at a dose of 2.0 mg per kg per day, while the control group received an equivalent volume (100 µL) of saline. Following the completion of modeling, mice were euthanized, and their hearts were harvested. Heart tissues were fixed overnight in 4% paraformaldehyde (PFA), dehydrated through a gradient series of sucrose solutions, and cut into frozen sections (thickness = 5–8 µm). The sections were mounted onto glass slides and incubated with the fluorescent chemosensor DO (10 µM) for 30 min at 37 °C. Fluorescence images were subsequently captured using a confocal microscope with an excitation wavelength of 488 nm and an emission detection range of 650–750 nm. For wheat germ agglutinin (WGA) staining, myocardial tissue sections were washed thrice with PBS, permeabilized with 0.1% Triton X-100 for 10 min, and blocked with 1% BSA for 30 min at room temperature. After removing the blocking solution, the sections were incubated with fluorescein isothiocyanate (FITC)-conjugated WGA working solution for 60 min at room temperature in the dark. Then, the cross-sectional area of cardiomyocytes was observed under a fluorescence microscope with an excitation wavelength of 488 nm and an emission wavelength of 500–550 nm. All animal experiments included five mice per group, and quantitative data are expressed as mean ± SEM. All animal experiments were approved by the Laboratory Animal Ethics Committee of the Affiliated Hospital of Chengdu University of Traditional Chinese Medicine (Ethics approval number: 2024DL-027).

### Statistical analyses

Data are the mean ± S.D. from at least five independent experiments. Significance between groups was assessed by the Student's *t*-test or one-way ANOVA with Tukey's test using Prism (GraphPad, La Jolla, CA, USA). *p* < 0.05 was considered significant.

## Results and discussion

### Design and synthesis of fluorescent chemosensors

**Scheme 1:**
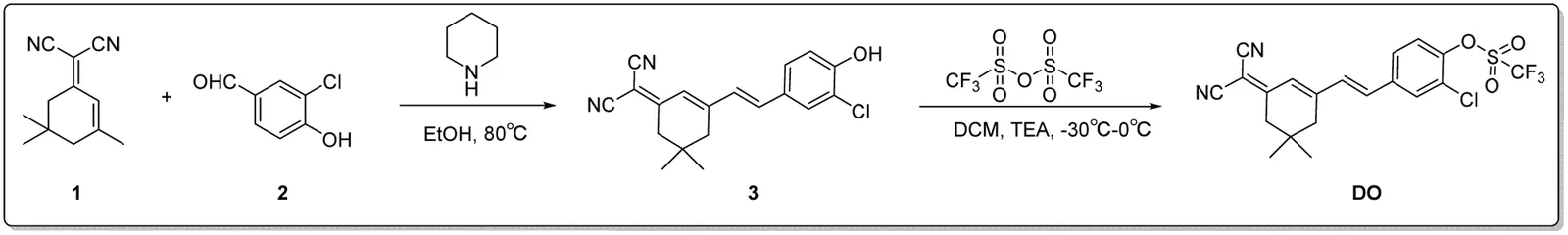
The synthesis of fluorescent chemosensor DO.

**Scheme 2:**
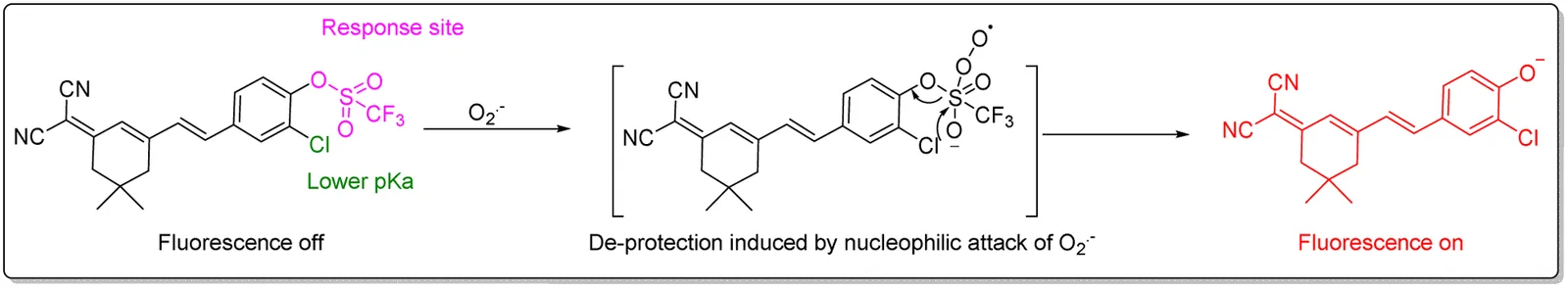
The proposed response mechanism of the fluorescent chemosensor DO toward O_2_˙^−^.

For the fluorophore scaffold, we selected dicyanoisophorone. This fluorophore possesses excellent biocompatibility, red emission, and a large Stokes shift, effectively avoiding self-absorption, fluorescence quenching, and biological toxicity.^28,29^ The recognition group for O_2_^˙−^ is trifluoromethanesulfonate. It is eliminated *via* nucleophilic attack by O_2_˙^−^, thereby exposing a phenolic hydroxyl group on the fluorophore. This specific reaction site has been widely adopted since it was first reported in 2018.^30^ Furthermore, a chlorine atom was introduced at the *ortho* position of the phenol to lower the overall _p_*K*_a_ of the molecule. This modification favors the formation of a phenolate anion, which has a stronger electron-donating capability after the reaction, thereby enhancing the fluorescence intensity. The detailed synthetic route and sensing mechanism are illustrated in Scheme 1. The proposed response mechanism of fluorescent chemosensor DO toward O_2_˙^−^ is shown in Scheme 2.

### Spectroscopic properties and optical response to O_2_˙^−^

**Fig. 1:**
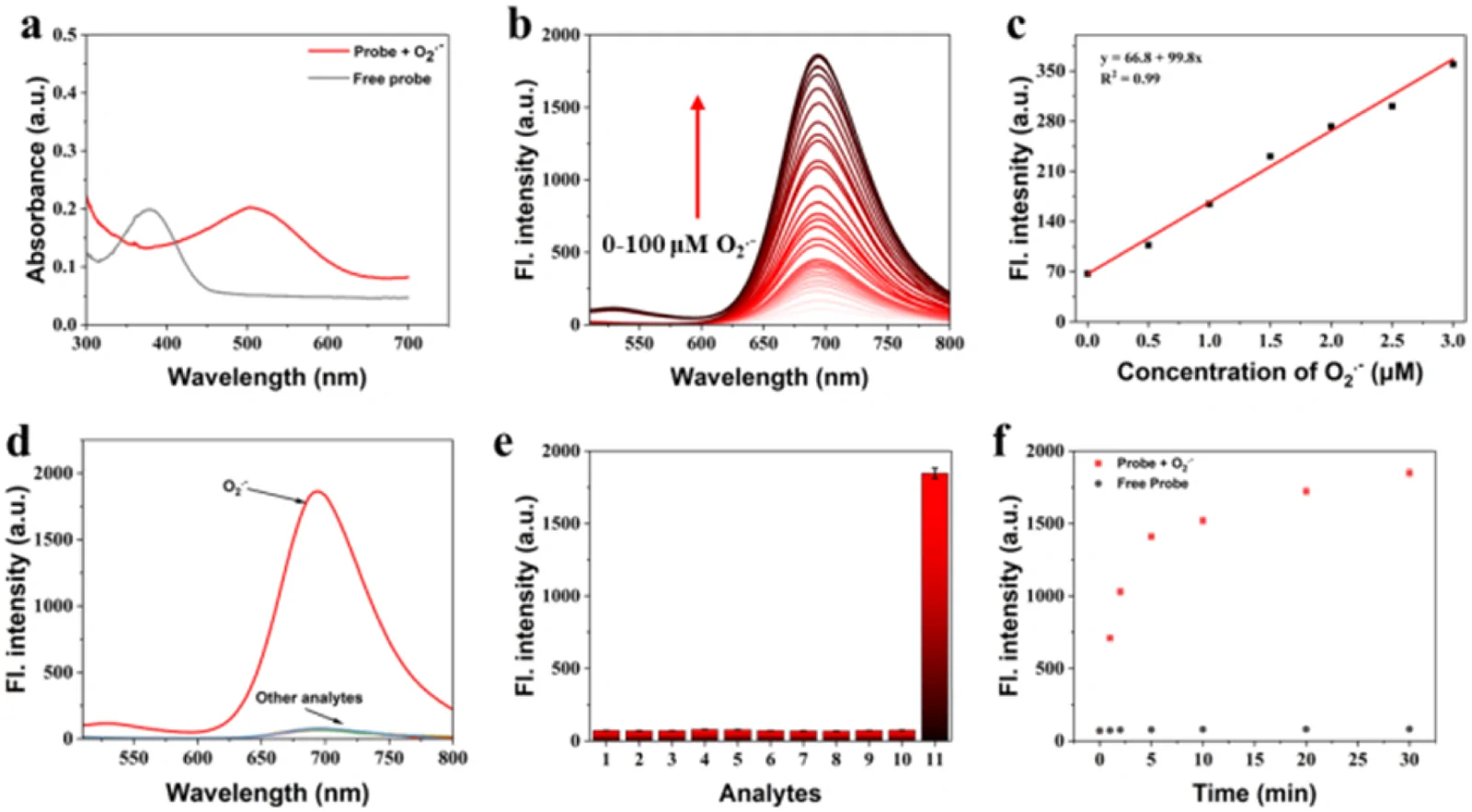
(a) Absorption spectra of chemosensor DO in the presence and absence of O_2_˙^−^. (b) Fluorescence spectra of chemosensor DO (10 µM) with O_2_˙^−^ (0–100 µM). (c) Linear relationship between the fluorescence intensity of chemosensor DO (10 µM) and O_2_˙^−^ concentration (0–2 µM). (d) Fluorescence spectra of chemosensor DO (10 µM) with each analyte. (1), blank; (2), H_2_O_2_; (3), HClO; (4), ONOO^−^; (5), NO; (6), .OH; (7), H_2_S; (8), Cys; (9), GSH; (10), Hcy; (11), ascorbate; (12), NADPH; (13), Na^+^; (14), K^+^; (15), Ca^2+^; (16), Mg^2+^; (17), Fe^2+^; O_2_˙^−^. The concentration of all analytes was set to 100 µM. (e) Fluorescence intensity at 698 nm of chemosensor DO (10 µM) in the presence of O_2_˙^−^ and other relevant species. (f) Fluorescence intensity of chemosensor DO (10 µM) in the presence of O_2_˙^−^ (100 µM) concentrations as a function of reaction time.

With the O_2_˙^−^ fluorescent chemosensor DO in hand, the first step was to investigate whether the fluorescent chemosensor could respond to O_2_˙^−^. O_2_˙^−^ was provided by potassium superoxide dissolved in DMSO. The test system was set as 10 mM PBS (containing 30% DMSO, pH = 7.4). Initially, as seen in Fig. 1a, the fluorescent chemosensor solution exhibited an absorption peak at 440 nm. Upon exposure to O_2_˙^−^, the fluorescent chemosensor DO showed an obvious red shift, with the maximum absorption wavelength moving to 500 nm. As seen in Fig. 1b, with regard to the fluorescence emission spectrum, upon excitation at 500 nm, the fluorescent chemosensor DO initially displayed negligible fluorescence intensity. With the addition of O_2_˙^−^, the fluorescence intensity increased sharply at 698 nm, resulting in a final enhancement of 25-fold. Meanwhile, the response of the fluorescent chemosensor to O_2_˙^−^ (0–2 µM) showed a good linear relationship (Fig. 1c). Meanwhile, we mixed DO with 1 equivalent of O_2_˙^−^, and after 10 s, we performed ultra-high-resolution mass spectrometry (HRMS). As seen in Fig. S7, the peaks corresponding to DO ([M–H]^−^ = 455.0422; the calculated value was 455.0522), and the deprotected dye were both detected in the same sample ([M–H]^−^ = 323.0934; the calculated value was 323.1029). This confirmed that DO responds to O_2_˙^−^ through deprotection to expose the hydroxyl group of the dye. Notably, the fluorescence quantum yield of the fluorescent chemosensor after the reaction reached 66.7%, and the limit of detection (LOD) was determined to be 93 nM. These results indicated that the fluorescent chemosensor DO possessed excellent responsiveness toward O_2_˙^−^. The molar extinction coefficient of DO was determined to be 29 900 M^−1^ cm^−1^, and it increased to 41 700 M^−1^ cm^−1^ after complete reaction with O_2_˙^−^. The fluorescence quantum yield of DO was measured to be 9.55% in its unreacted state, whereas after complete reaction with O_2_˙^−^, it increased to 68.83%, reflecting a significant enhancement and high-brightness signal reporting capability.

Next, to evaluate whether the fluorescent chemosensor DO could function as a sensor for O_2_˙^−^ in complex biological systems, its responses to other ROS similar to O_2_˙^−^, such as H_2_O_2_,^.^OH, ONOO^−^, nitric oxide (NO), and hypochlorous acid (HClO), were also investigated. We also studied nucleophiles such as glutathione (GSH), cysteine (Cys), homocysteine (Hcy), and hydrogen sulfide (H_2_S), biological reductants such as ascorbate and nicotinamide adenine dinucleotide phosphate hydrogen (NADPH), and metal ions such as Na^+^, K^+^, Ca^2+^, Mg^2+^ and Fe^2+^. None of these species could trigger the deprotection of the fluorescent chemosensor DO to form the red fluorophore or induce a red shift in absorption, indicating the excellent selectivity of the fluorescent chemosensor.

Meanwhile, the response capability of DO under different pH conditions was tested. As seen in Fig. S1, the fluorescent chemosensor without O_2_˙^−^ showed weak fluorescence at pH 4–10. After reacting with O_2_˙^−^, the fluorescence remained weak under acidic conditions, whereas it increased significantly under neutral and alkaline conditions (with an enhancement fold greater than 25). Therefore, the fluorescent chemosensor DO appeared to be suitable for detecting O_2_˙^−^ in the neutral microenvironment of cells. Finally, the response kinetics of the probe were carefully investigated. Upon exposure to O_2_˙^−^, the fluorescence intensity of chemosensor DO rapidly increased by approximately 10-fold within 1 min and reached a *plateau* at around 30 min. In contrast, in the absence of O_2_˙^−^, the fluorescence intensity of the chemosensor DO remained almost unchanged over 30 min (Fig. 1f). Collectively, these data demonstrated that the fluorescent chemosensor DO exhibited high selectivity and strong anti-interference capability toward O_2_˙^−^.

### Cytotoxicity and fluorescence imaging

Encouraged by the favorable *in vitro* spectral response of the fluorescent chemosensor, confocal fluorescence imaging was subsequently performed in living cells. Initially, the cytotoxicity of the fluorescent chemosensor DO was evaluated. Even after incubation with a high concentration of fluorescent chemosensor (50 µM) for 12 h, the cell mortality remained below 20%, indicating low biological toxicity of the fluorescent chemosensor (Fig. S2). Photostability tests were subsequently carried out to ascertain if DO was suitable for cell imaging. DO exhibited less than 10% decrease in absorbance within 30 min under LED irradiation (10 W) at 365 nm, confirming its high photostability (Fig. S8).

**Fig. 2:**
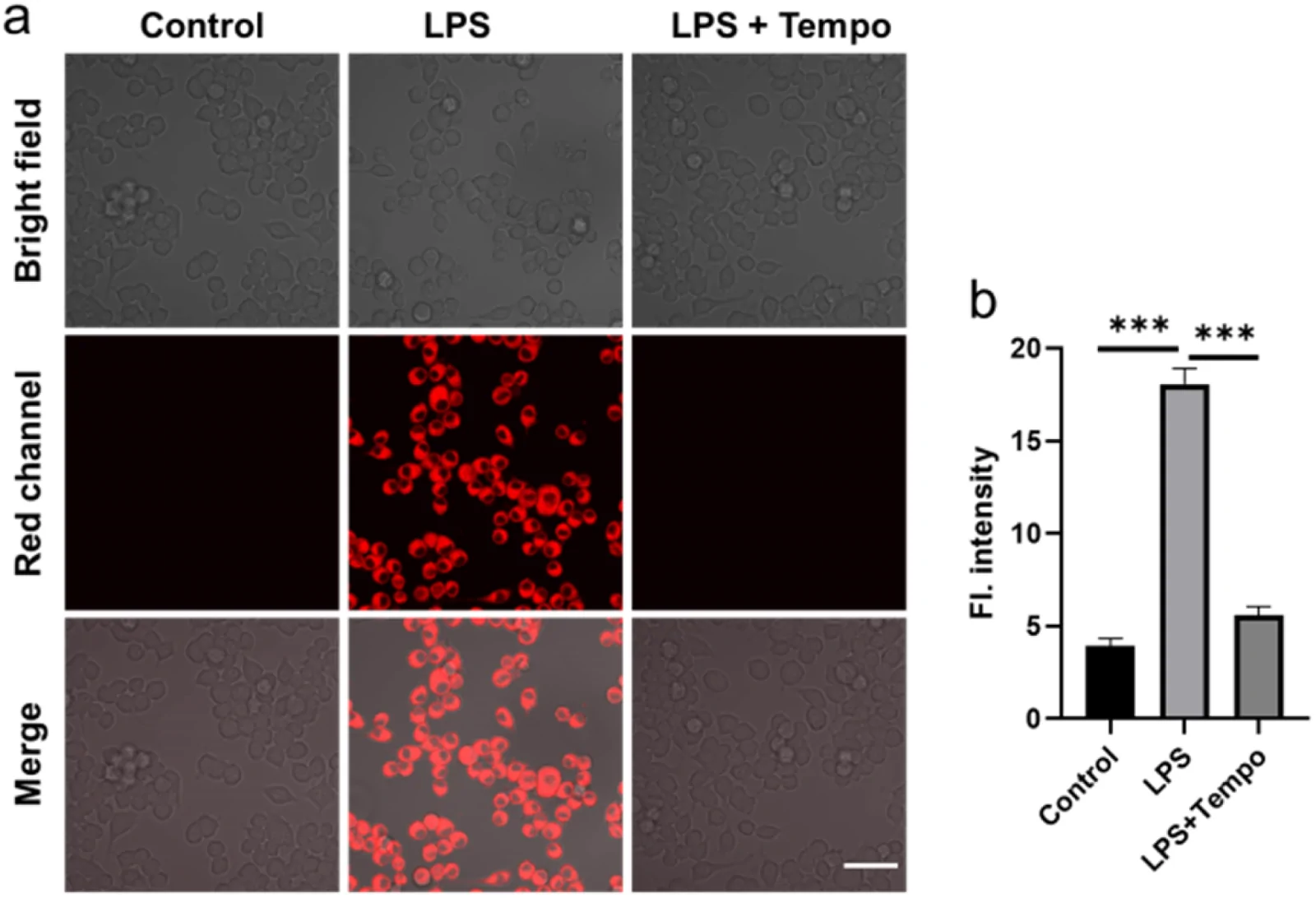
(a) Confocal imaging of the chemosensor DO responding to endogenous O_2_˙^−^ in living RAW264.7 cells. From top to bottom: bright field, red channel, merge of red channel and bright field. From left to right: control, LPS (1 µg mL^−1^) treated for 12 h, LPS (1 µg mL^−1^) pretreated for 12 h and then Tempo (10 µM) for 1 h. (b) Relative fluorescence intensity of live cell imaging. Scale bar = 20 µm. *λ*_em_ = 650–750 nm, *λ*_ex_ = 488 nm. *n* = 5.

To verify the responsiveness of the fluorescent chemosensor in live cells, we initially employed RAW264.7 macrophages as a model cell line, as shown in Fig. 2a and b, using lipopolysaccharide (LPS) as an agonist to stimulate the endogenous generation of O_2_˙^−^. The first group of cells (incubated with the fluorescent chemosensor DO only) exhibited negligible red fluorescence under laser excitation of 488 nm. In contrast, the second group, which was pretreated with LPS for 12 h, displayed intense red fluorescence, with the intensity being approximately 4.5-fold higher than that of the control group. Conversely, in the third group, the addition of the radical scavenger 2,2,6,6-tetramethylpiperidinooxy (TEMPO) 1 h prior to incubation with fluorescent chemosensor rapidly depleted the intracellular O_2_˙^−^, resulting in a significant decrease in fluorescence. Collectively, these results indicated that the fluorescent chemosensor DO could sensitively reflect the fluctuations in intracellular O_2_˙^−^ concentration.

### Cellular co-localization

**Fig. 3:**
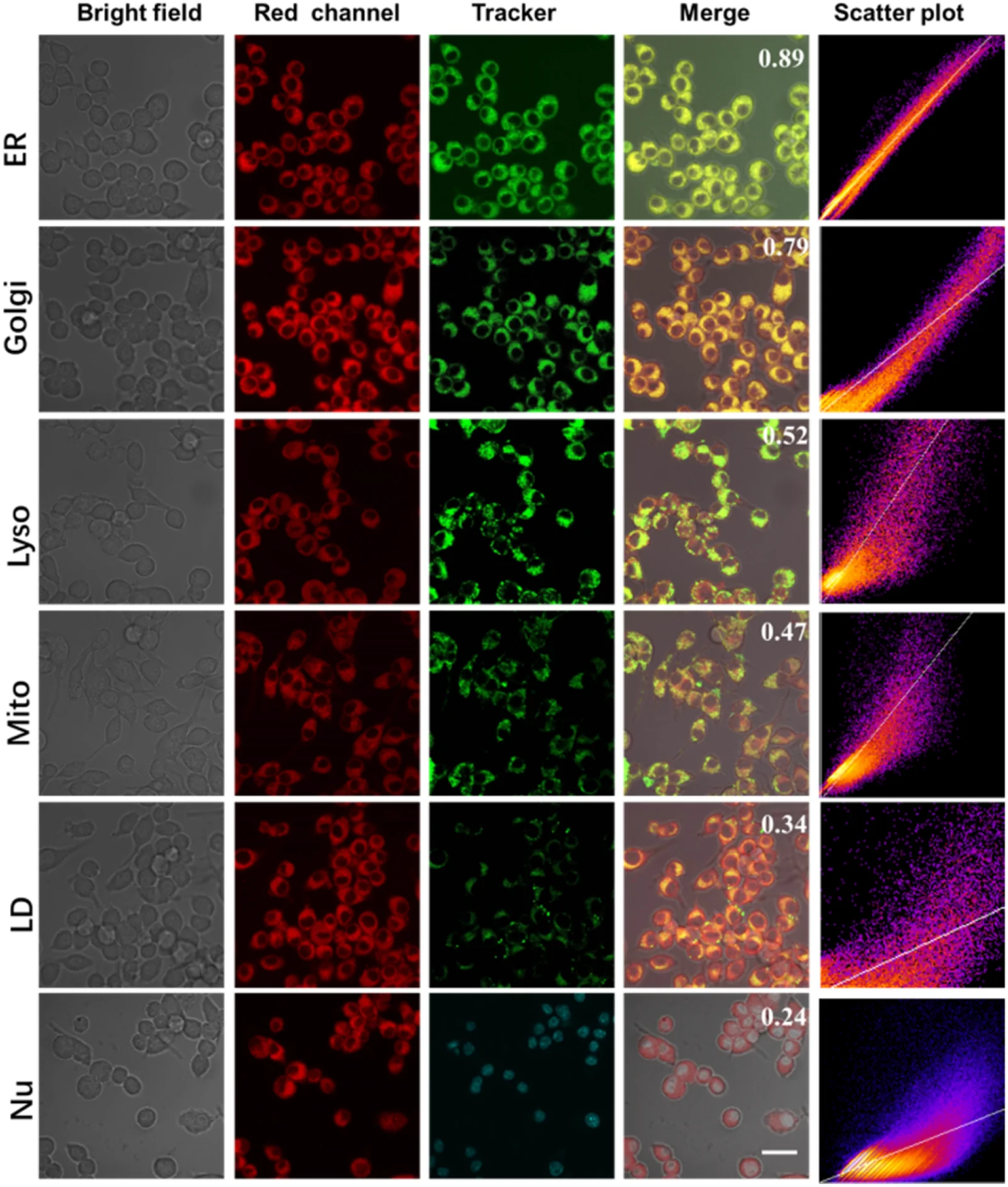
Co-localization imaging of chemosensor DO. From left to right: bright field, red channel, organelle tracker channel, merge image, scatter plot. From top to bottom: endoplasmic reticulum (ER), Golgi body, lysosome, mitochondrion, lipid droplet, nucleus. Red channel: *λ*_em_ = 650–750 nm, *λ*_ex_ = 488 nm. Green channel: *λ*_em_ = 500–550 nm, *λ*_ex_ = 488 nm. Especially, nucleus tracker: *λ*_em_ = 420–460 nm, *λ*_ex_ = 405 nm. Scale bar = 20 µm. Chemosensor DO (10 µM) pretreated with LPS (1 µg mL^−1^) for 12 h, concentration of each tracker was 100 nM.

In recent years, subcellular targeting of small-molecule fluorescent chemosensors has been increasingly emphasized because it is crucial for the spatial resolution of imaging. Therefore, we conducted further investigations into the subcellular targeting of fluorescent chemosensor DO. Commercial organelle trackers (green or blue) were used to stain specific organelles as standard references, and cells were pretreated with LPS to ensure the fluorescent chemosensor was maximally activated within the cells. As shown in Fig. 3, the Pearson's co-localization coefficient between the fluorescent chemosensor DO and commercial trackers was: 0.89 for endoplasmic reticulum (ER); 0.47 for mitochondrion (Mito); 0.52 for lysosome (Lyso); 0.79 for Golgi body (Golgi); 0.34 for lipid droplet (LD); 0.24 for and nucleus (Nu). These findings indicated that the fluorescent chemosensor DO was distributed throughout the cytoplasm and various organelles, with a higher selectivity for the ER.

### Cellular diagnosis of cardiac hypertrophy

**Fig. 4:**
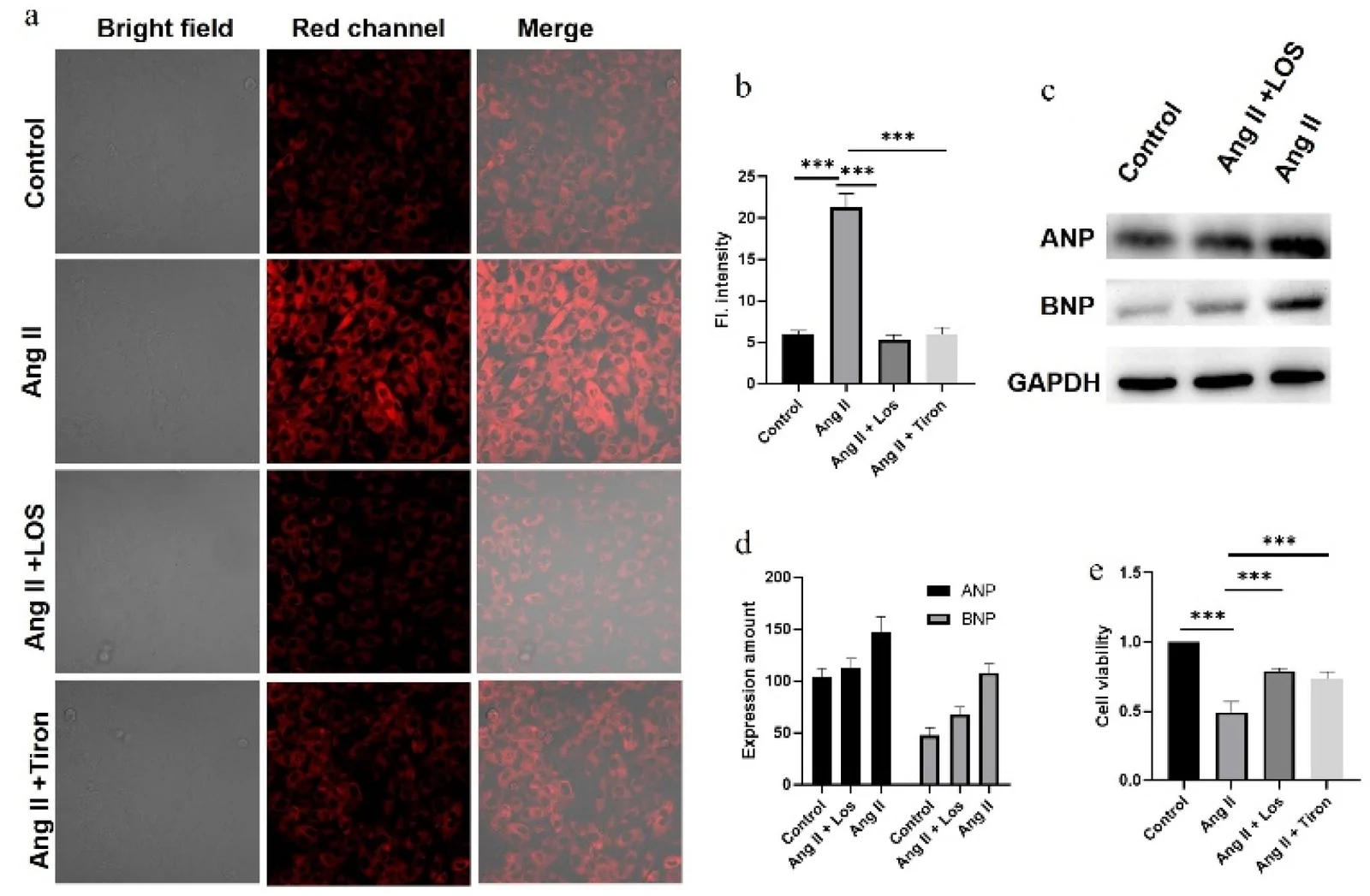
(a) Confocal imaging of the chemosensor DO responding to endogenous O_2_˙^−^ in living H9c2 cells. From top to bottom: control, Ang II (100 nM) treated for 48 h, Ang II (100 nM) pretreated for 48 h and then Los (10 µM) for 24 h, Ang II (100 nM) pretreated for 48 h and then Tiron (10 µM) for 12 h. From left to right: bright field, red channel, merge of red channel and bright field. (b) Relative fluorescence intensity of live H9c2 cell imaging. (c) Western blots of ANP and BNP protein expression to assess cardiomyocyte hypertrophy. Ang II (100 nM) treated for 48 h, Ang II (100 nM) pretreated for 48 h and then Los (10 µM) for 24 h. (d) Relative protein levels analyzed for ANP and BNP. (e) Cell viability under each treatment through the CCK-8 assay. Scale bar = 20 µm. *λ*_em_ = 650–750 nm, *λ*_ex_ = 488 nm. *n* = 5.

Next, the fluorescent chemosensor was incubated with H9c2 rat cardiomyocytes for confocal imaging. As illustrated in Fig. 4a and b, weak fluorescence was observed in the first group (cells incubated with the fluorescent chemosensor only) under laser excitation (488 nm). In the second group, cells were pretreated with Ang II (100 nM) for 48 h to stimulate the intracellular production of ROS. Consequently, bright-red fluorescence was observed, with the fluorescence intensity increasing approximately 4.8-fold compared with that in the untreated control group. Interestingly, when cells treated with Ang II were pretreated with losartan (Los, an angiotensin II type-1 (AT1) receptor antagonist), the fluorescence intensity decreased even further, dropping to almost the same level as that in the control group.

Collectively, these data demonstrated that the fluorescent chemosensor DO could be applied to myocardial-related cells. It sensitively sensed the fluctuations of intracellular O_2_˙^−^ and innovatively visualized the microenvironmental changes in myocardial cells under Ang-II stress through variation in the fluorescence signal. To investigate the intracellular metabolic changes in cardiomyocytes induced by increased O_2_˙^−^ levels, protein extracts were prepared from the four fluorescent groups for Western blotting. As seen in Fig. 4c and d, the expression of atrial natriuretic peptide (ANP) and brain natriuretic peptide (BNP), biomarkers indicative of hypertrophic injury, was measured. Treatment with Ang II significantly increased the production of ANP and BNP, suggesting myocardial hypertrophy. However, Los attenuated this increase, indicating an improvement in hypertrophy. Meanwhile, cell viability was assessed using the Cell Counting Kit-8 (CCK-8) assay. We found that 48-h treatment with Ang II reduced cardiomyocyte viability by 50%. In contrast, treatment with Los restored cell viability to approximately 80%. Collectively, these results demonstrated that the increase of ROS in cardiomyocytes was accompanied by myocardial hypertrophy, which may further lead to cell death if not intervened in a timely manner.

### Analyses of cardiac sections from hypertrophic hearts

Finally, to validate the feasibility of the fluorescent chemosensor DO for *in vivo* applications, a mouse model of cardiac hypertrophy was established using Ang II administered at a dose of 2.0 mg per kg per day. After sacrificing the mice, heart tissues were harvested and cut for frozen sectioning. The frozen heart slices were then incubated with fluorescent chemosensor DO.

**Fig. 5:**
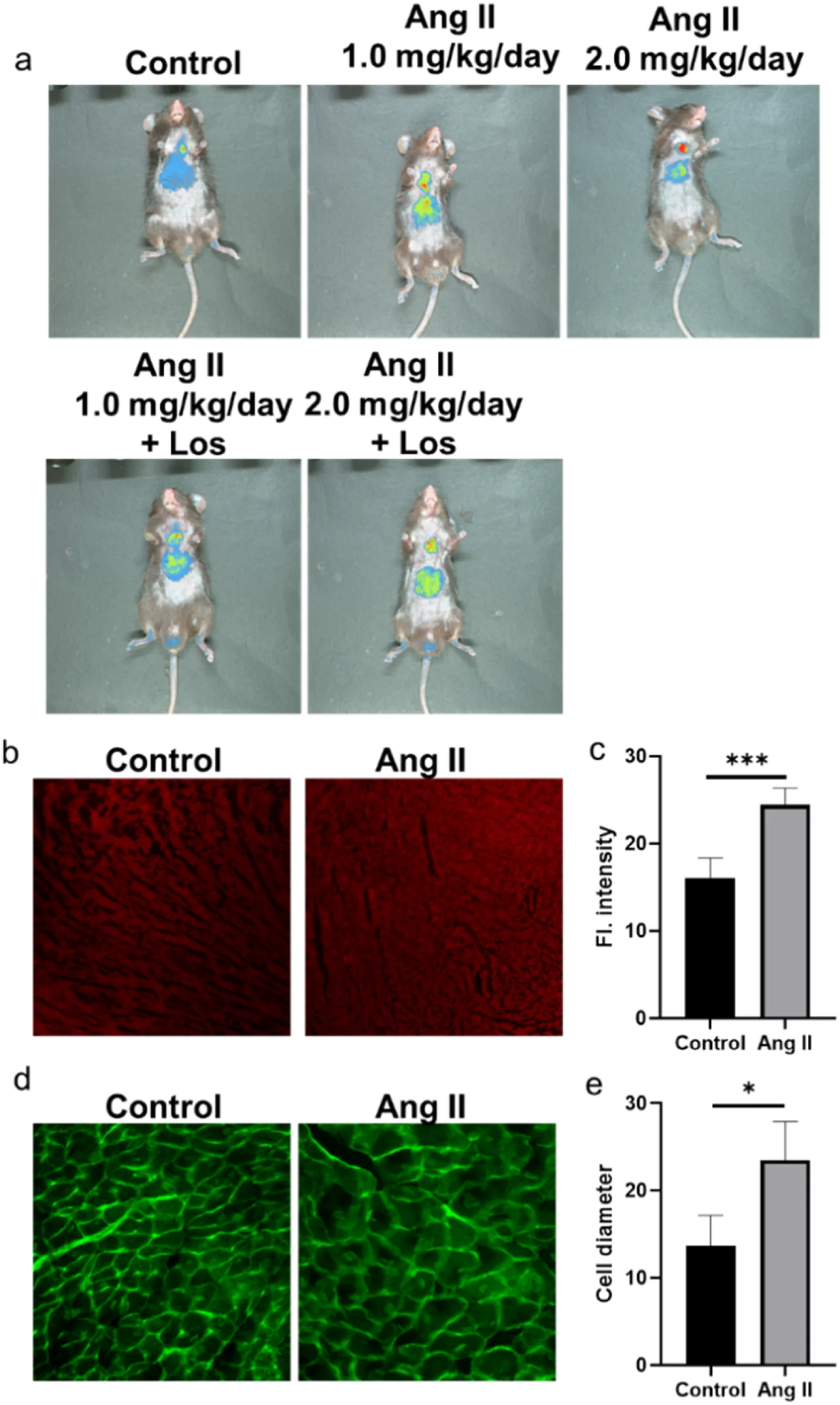
(a) *In vivo* imaging for different groups of mice. *λ*_em_ = 650–750 nm, *λ*_ex_ = 488 nm. (b) Fluorescence imaging of cardiac hypertrophy sections by treatment of chemosensor DO (10 µM) for 30 min under 37 °C. (c) Fluorescent intensity of cardiac hypertrophy sections. *λ*_em_ = 650–750 nm, *λ*_ex_ = 488 nm. (d) WGA staining of cardiac hypertrophy sections. (e) Measurement of average cell diameter from (d) *λ*_em_ = 500–540 nm, *λ*_ex_ = 488 nm. *n* = 5.

The myocardial fluorescence intensity in the Ang II-induced hypertrophy group was significantly higher than that in the control group, with an intensity being approximately 2.5-fold greater. This indicated a robust burst of O_2_˙^−^ in the hearts of mice with cardiac hypertrophy. Concurrently, WGA staining was performed to assess cardiomyocyte size. WGA specifically recognizes *N*-acetylglucosamine and sialic acid residues on the cardiomyocyte membrane. Upon incubation with fluorescently labeled WGA, the cell membrane is specifically stained, clearly delineating the boundaries of individual cardiomyocytes. The cardiomyocyte cross-sectional area in the hypertrophy group was about 1.3 times greater than that in the normal control group. Collectively, these data demonstrated that DO could detect elevated O_2_˙^−^ levels in hypertrophic mouse cardiac tissue to reflect myocardial hypertrophy-related oxidative stress (Fig. 5).

## Conclusions

We designed and synthesized a fluorescent chemosensor DO targeting intracellular O_2_˙^−^. Chemosensor DO exhibited several advantages, including a rapid response (approximately 10-fold fluorescence enhancement within 1 min), high selectivity, and a LOD (93 nM). This probe was applied to evaluate the concentration of O_2_˙^−^ in a cellular model of cardiac hypertrophy. Furthermore, Western blotting revealed the progression of cardiac hypertrophy to be associated with an O_2_˙^−^ burst, which could be inhibited by Los. Finally, in heart tissue sections from hypertrophic mice, chemosensor DO visualized the elevated O_2_˙^−^ levels. The observed trends were consistent with the results obtained using classical WGA staining. Therefore, chemosensor DO could serve as a fluorescent imaging tool to monitor O_2_˙^−^ accumulation in cell and tissue models of myocardial hypertrophy.

## Author contributions

F. G. and Y. L. designed the study and the experiments. Q. C. collected data and was responsible for interpretation. Y. D. directed and coordinated the whole process and oversaw the project. F. G. and Y. L. drafted the manuscript. All authors have agreed to the published version of the manuscript.

## Conflicts of interest

There are no conflicts of interest to declare.

## Supplementary Material

RA-OLF-D6RA00466K-s001

RA-OLF-D6RA00466K-s002

## Data Availability

The original data presented in this study are included in the article/supplementary information (SI). Further inquiries can be directed to the corresponding author (Yasheng Deng). Supplementary information: synthesis and characterization of probe DO, supplementary spectroscopic and cell‑based experimental data, and a comparison table of reported related probes. See DOI: https://doi.org/10.1039/d6ra00466k.
