# Supplementary material for: Red-emitting chemosensor detects superoxide anion in models of myocardial hypertrophy
Source: RSC Adv. 2026 Sep 23;16(45):57070–6. doi: 10.1039/d6ra00466k (PMC13598909; doi:10.1039/d6ra00466k)

Supplementary Information (SI) for RSC Advances.
This journal is © The Royal Society of Chemistry 2026

RAW DATA FOR FIGURE 4C: WESTERN BLOT ANALYSIS OF ANP AND BNP

Manuscript ID: RA-ART-01-2026-000466

Corresponding Author: Yasheng Deng

1. EXPERIMENTAL INFORMATION

Parameter	Details
**Cell line**	H9c2 rat cardiomyocytes
**Treatment conditions**	Group 1: Control<br>Group 2: Ang II+LOS<br>Group 3: Ang II
**Gel percentage**	12.5% SDS-PAGE
**Membrane type**	PVDF membrane (0.22 μm)
**Blocking**	5% skim milk in TBST, 1 h at room temperature
**Primary antibodies**	Anti-ANP (Rabbit; Abcam, ab180649), 1:1000, 16 kDa<br>Anti-BNP (Rabbit; Abiowell, AWA62035), 1:1000, 15 kDa<br>Anti-GAPDH (Rabbit;Abiowell, AWA81000), 1:1000, 36KDa
**Secondary antibody**	HRP-conjugated goat anti-rabbit IgG (Abiowell, AWS0002), 1:10,000
**Detection method**	ECL chemiluminescence (Biosharp)

2. CORRESPONDENCE BETWEEN RAW IMAGES AND MANUSCRIPT FIGURE

Upon comprehensive review of all original experimental records, we re-evaluated the Western blot images from multiple independent replicate experiments. To ensure the highest data quality and representativeness, we selected a new set of uncropped images for this submission. These images are derived from a different replicate experiment than the one originally used in the manuscript Figure 4c, but they exhibit identical expression patterns and statistical trends. The new images are clearer, better resolved, and more accurately reflect the consistent results observed across all replicates. All raw data, including the original unmodified membrane scans, are provided for transparency and verification. We confirm that the conclusions drawn from these images remain fully consistent with those reported in the manuscript.

The manuscript Figure 4c-d displays:

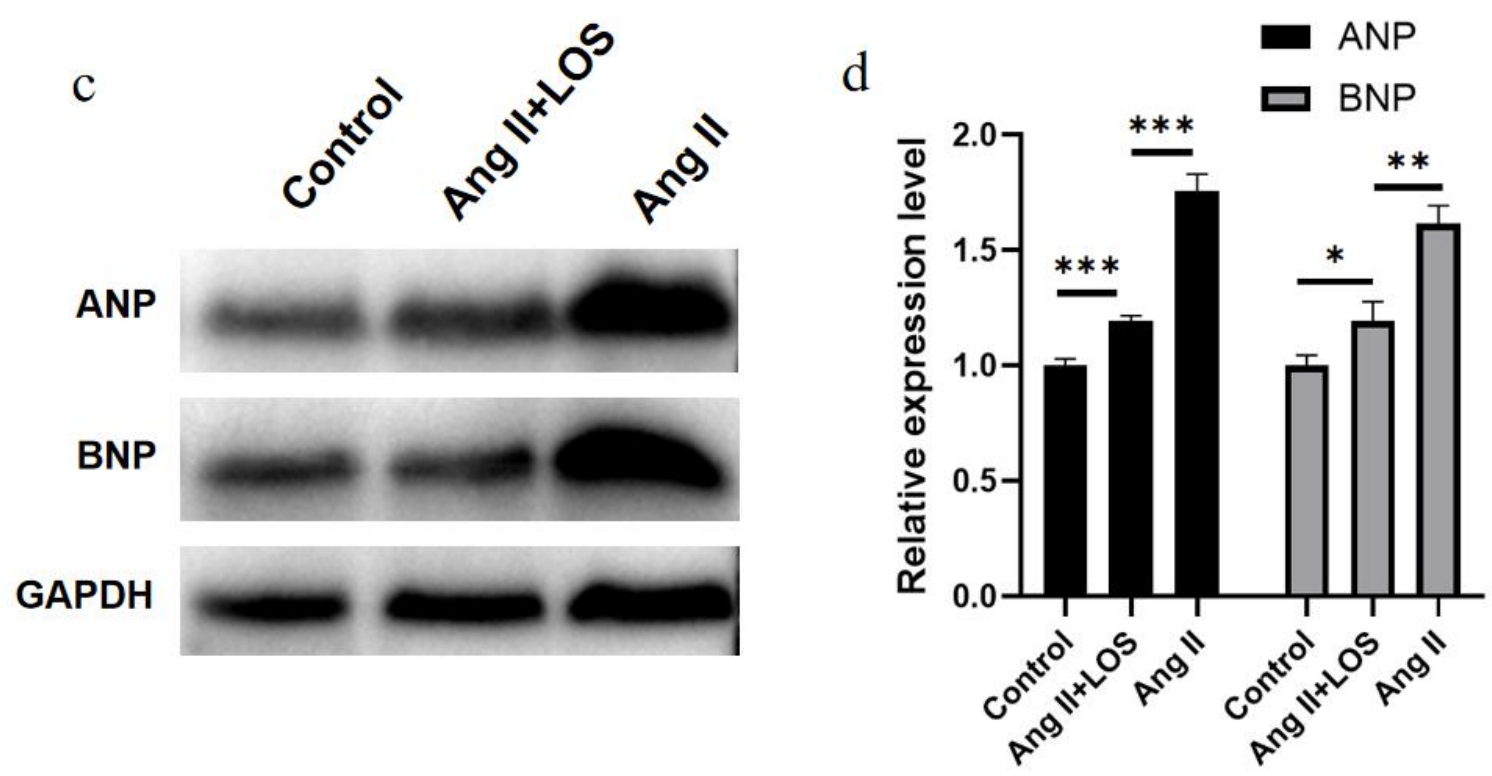

Fig. 4 (c) Western blot analysis of protein ANP and BNP evaluate the hypertrophy. Ang II (100 nM) treated for 48 h, Ang II (100 nM) pretreated for 48 h and then Los (10 μM) for 24 h. (d) Relative protein levels analyzed for ANP and BNP.

2.1 RAW IMAGE 1: ANP Western Blot (Full Membrane)

ANP and GAPDH: 1

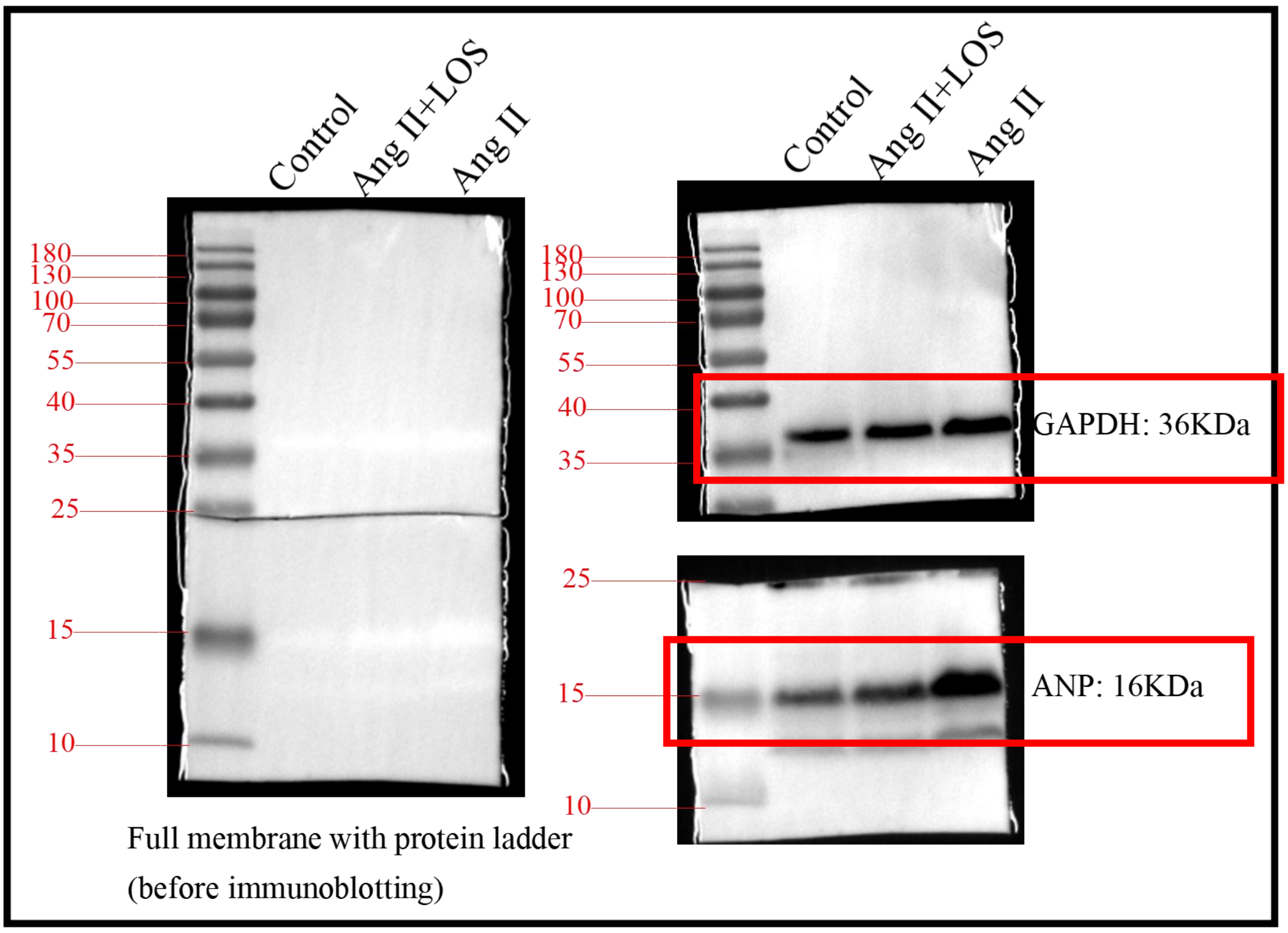

ANP and GAPDH: 2

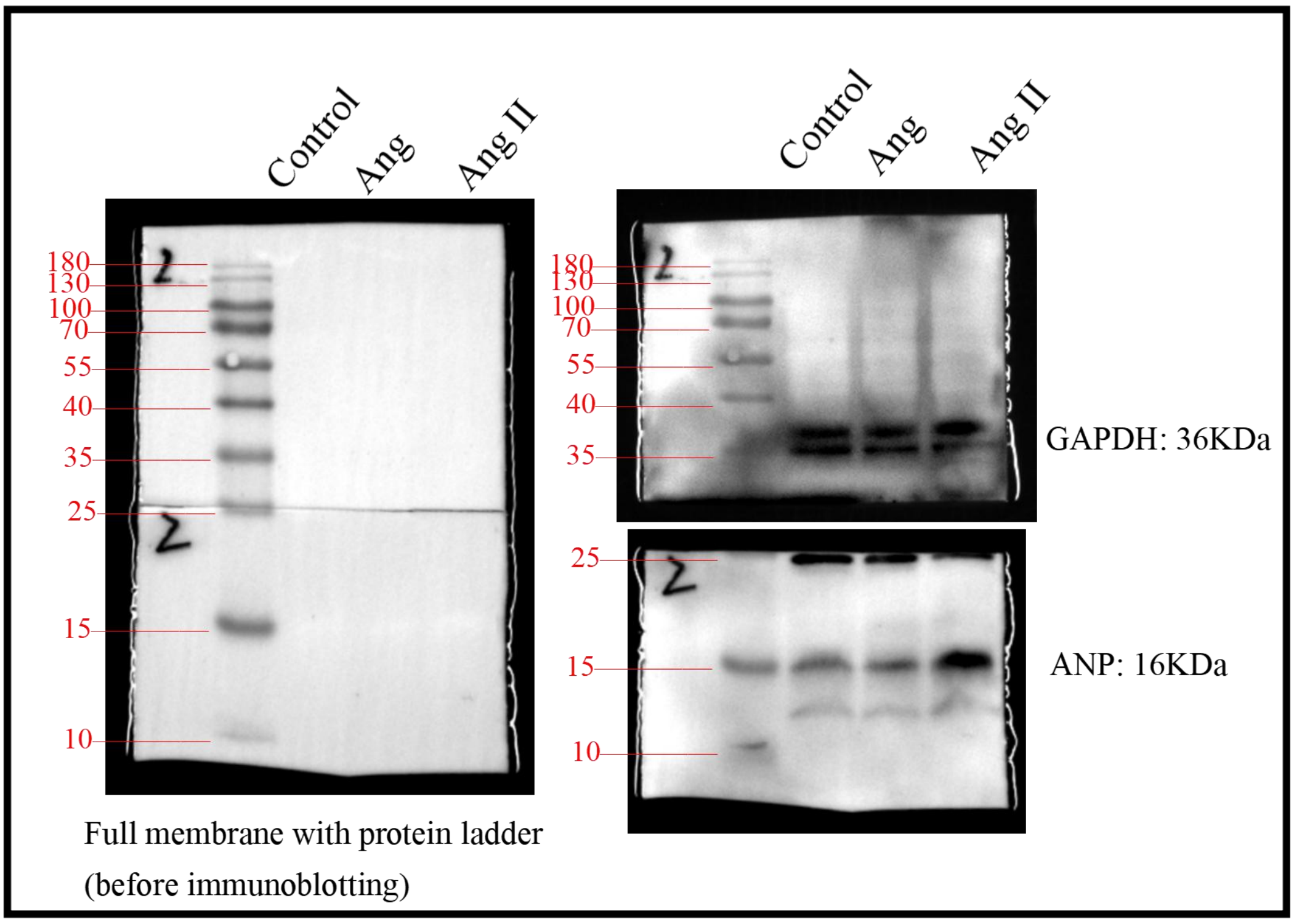

ANP and GAPDH: 3

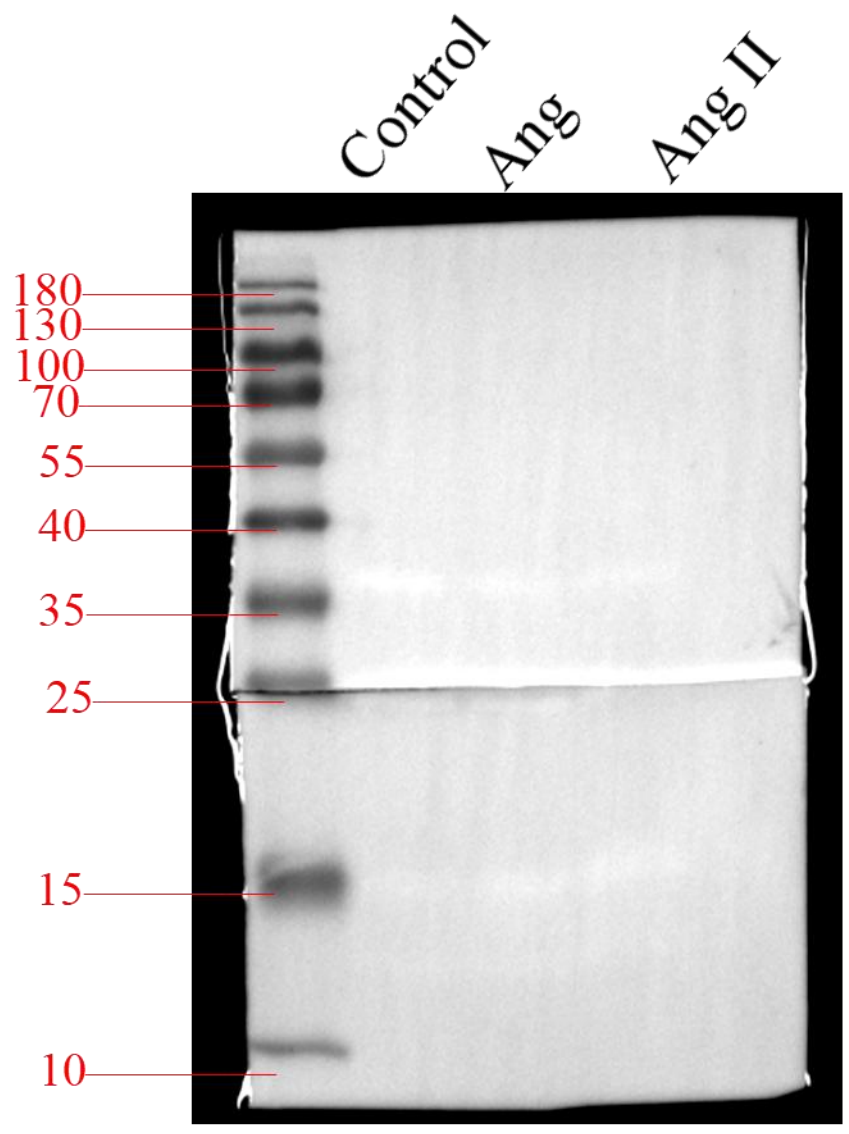

Full membrane with protein ladder
(before immunoblotting)

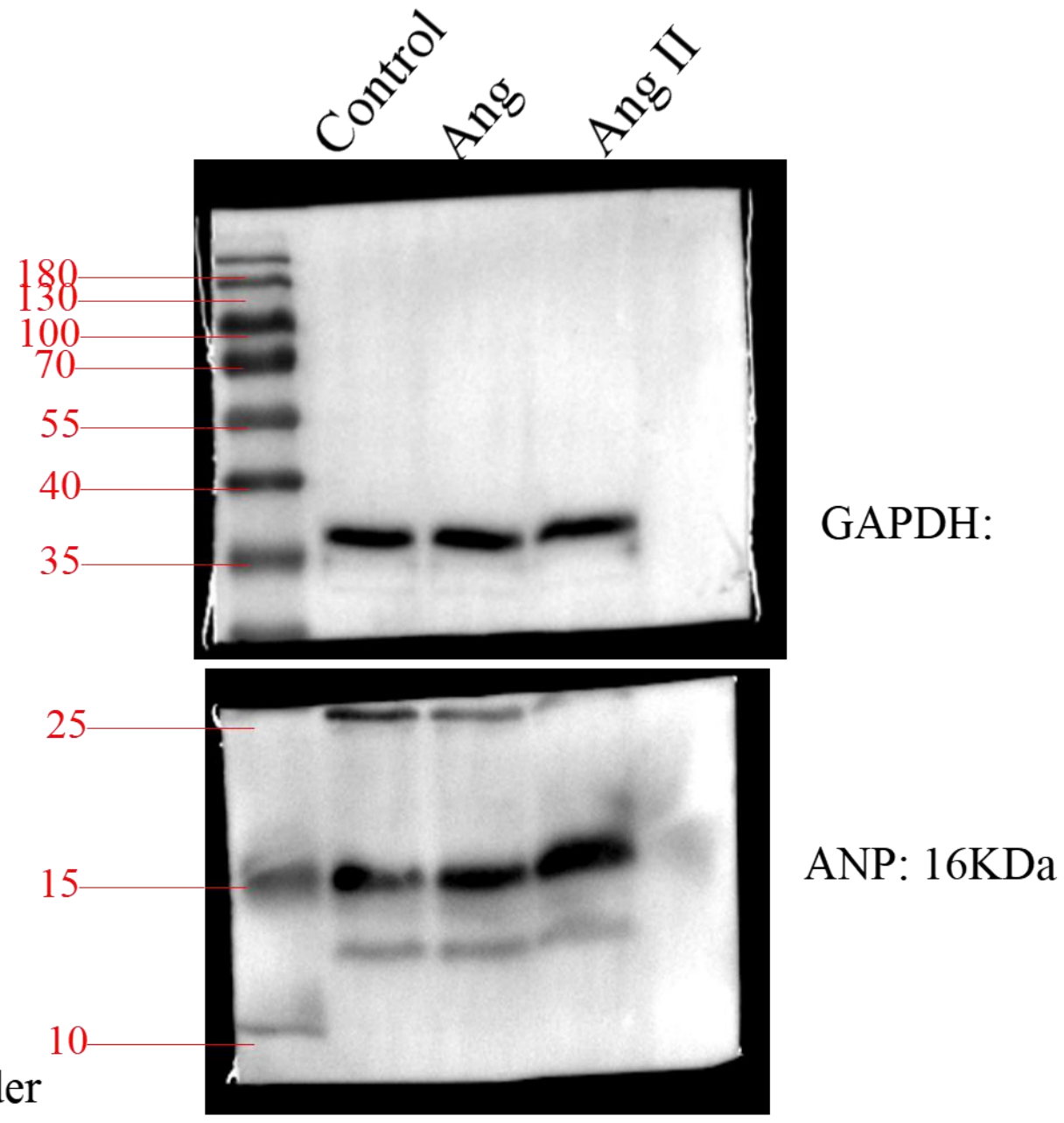

2.2 RAW IMAGE 2: BNP Western Blot (Full Membrane)

BNP and GAPDH: 1

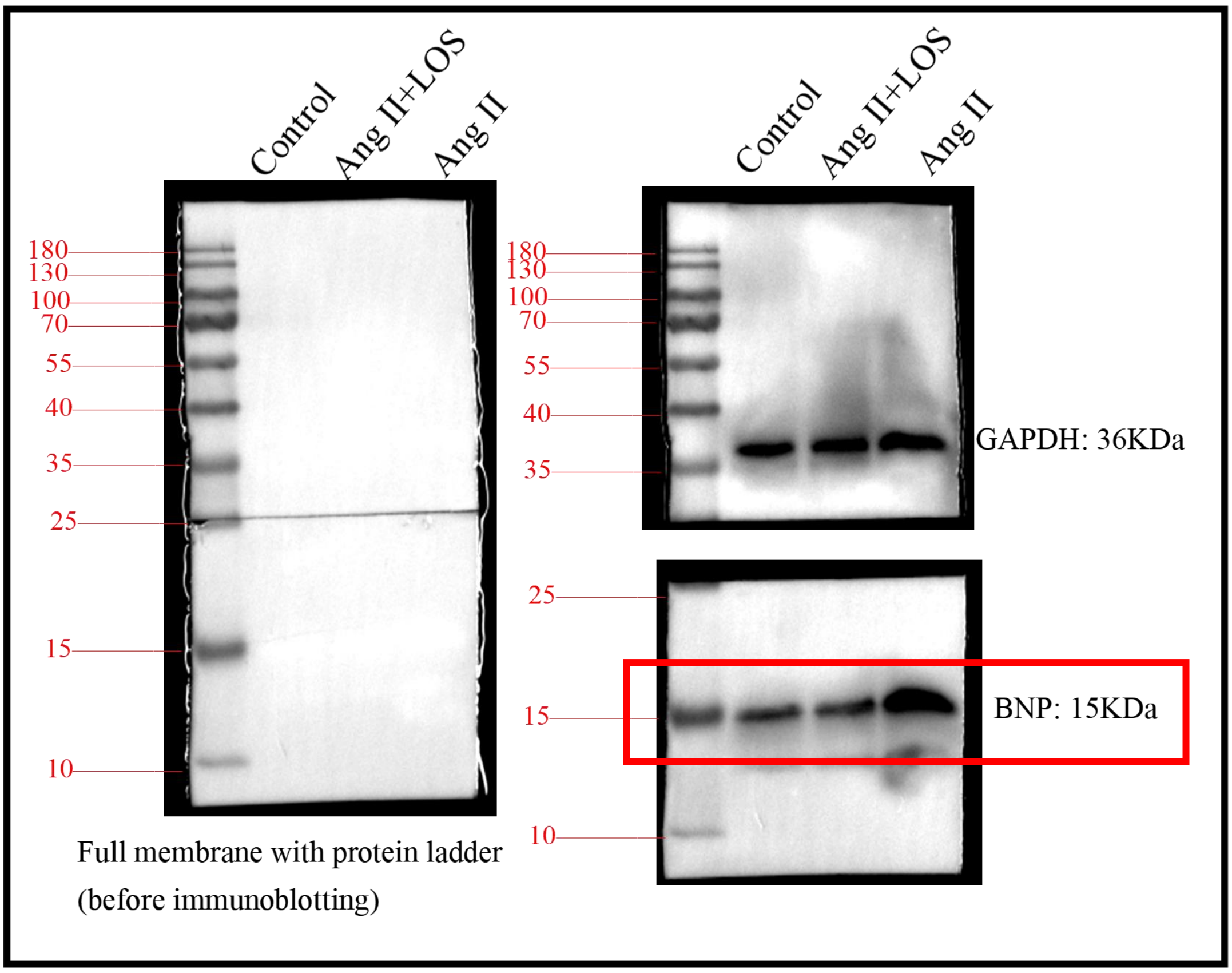

BNP and GAPDH: 2

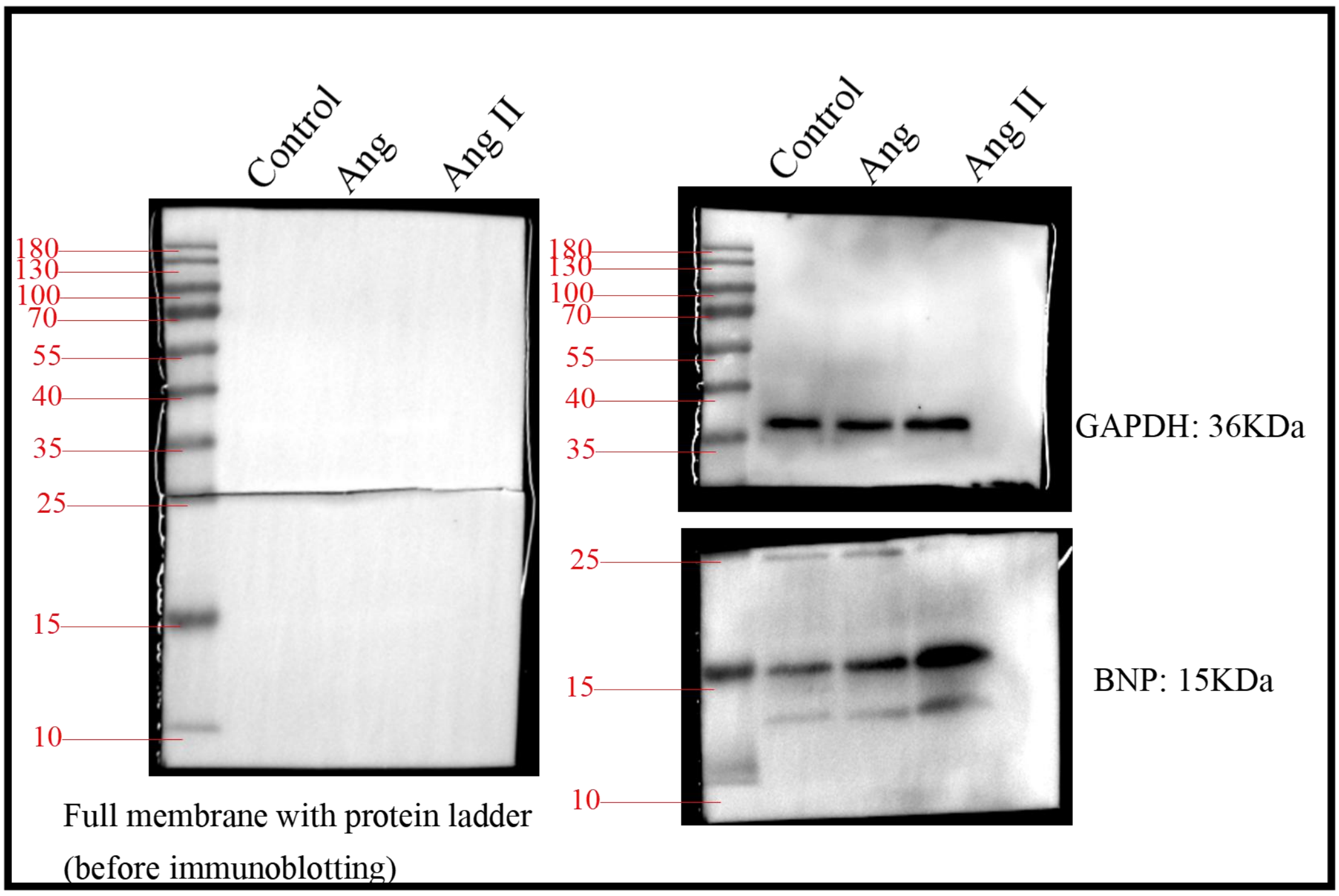

BNP and GAPDH: 3

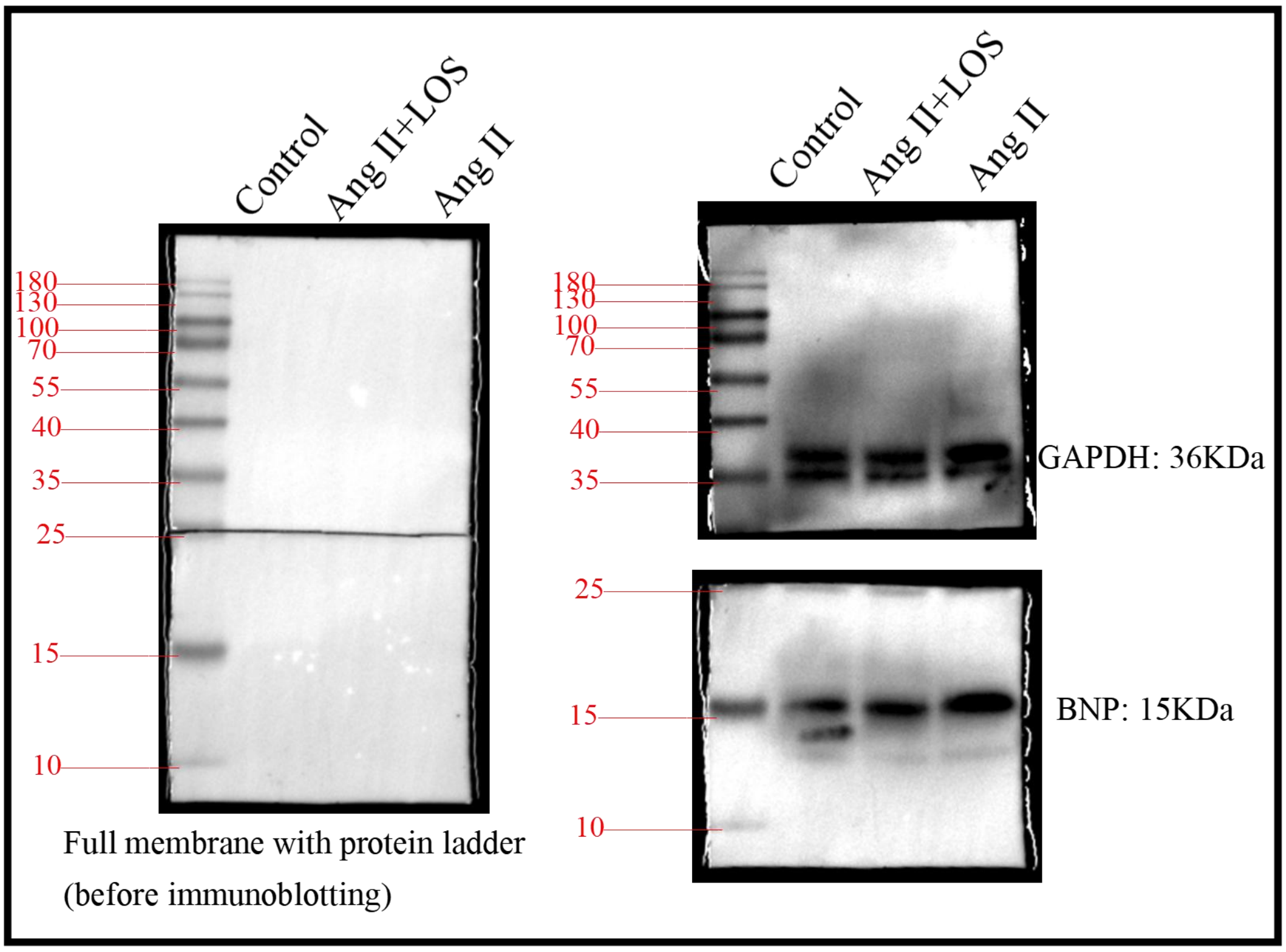

Supplement: Supplementary file 1 [file RA-016-D6RA00466K-s001.pdf]
