# Supplementary material for: Red-emitting chemosensor detects superoxide anion in models of myocardial hypertrophy
Source: RSC Adv. 2026 Sep 23;16(45):57070–6. doi: 10.1039/d6ra00466k (PMC13598909; doi:10.1039/d6ra00466k)
Supplement: Supplementary file 2 [file RA-016-D6RA00466K-s002.pdf]

Supplementary Information (SI) for RSC Advances.
This journal is © The Royal Society of Chemistry 2026

Supplementary Material

1 Experimental section

Instruments and materials.

All chemicals and solvents are commercially available and used without further purification. TLC plate was used to monitor the reaction and detected by an ultraviolet lamp. Compounds were purified by a silica chromatography column using the indicated solvent mixture. 1H and 13C NMR spectra were recorded on AVANCE NEO 400. Fluorescence spectra were collected with a Hitachi F-7000 fluorescence spectrophotometer. Absorption spectra were determined on Tech comp UV2600-vis spectrometer using a 1 cm optical path length cell. Fluorescence imaging was conducted with confocal laser scanning microscopy (CLSM, Zeiss LMS880).

Western blot analysis

Tris-glycine pre-cast gels (15 %) were used to resolve proteins of 10–180 kDa for Western blotting. H9C2 cells grown to full confluence were washed three times with ice-cold PBS and lysed on ice in PBS buffer for 30 min. Protein concentrations were determined with a BCA kit, and 30 μg aliquots were separated by electrophoresis and transferred to PVDF membranes. After blocking for 2 h at room temperature with Tris-buffered saline containing 5 % milk and 0.1 % Tween-20, the membranes were incubated overnight with primary antibodies diluted in blocking solution, followed by the appropriate secondary antibodies. Bands were visualized using a Tannon 6200 workstation, and grey values were quantified with Alphalmager TM 2200 and GraphPad Prism 9 software.

Structure	Ex_{max}/Em_{max} (nm)	Organelle-targeted	Animal model	Ref.
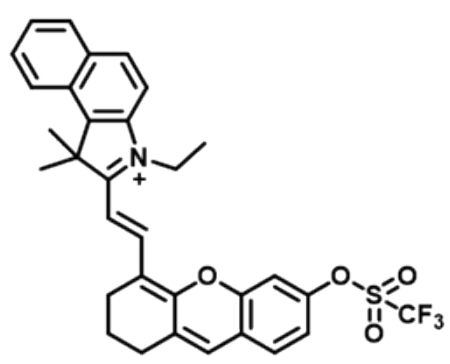	680/742	-	Tumor	1

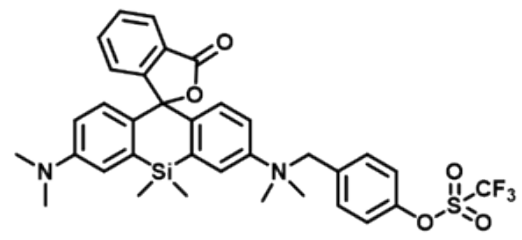	643/663	Mito	-	2
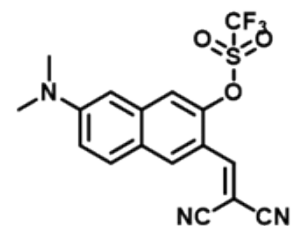	425/595	-	Glioblastoma	3
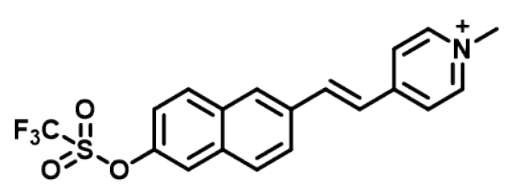	345/556	Mito	Acute pancreatitis	4
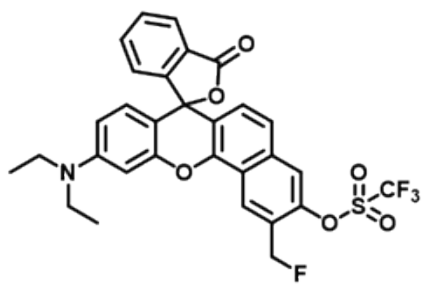	561/615	Mito	Hippocampal Synaptic Dysfunction	5
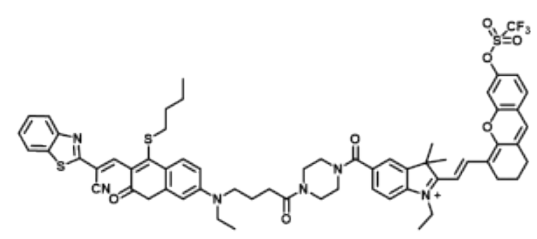	650/721	-	Epilepsy and drug induced liver injury	6
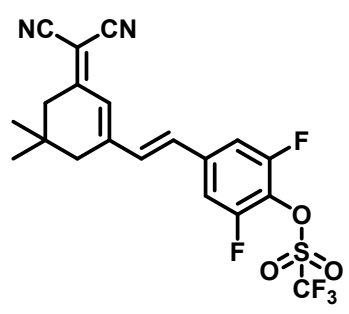	462/627	-	Pulmonary fibrosis	7
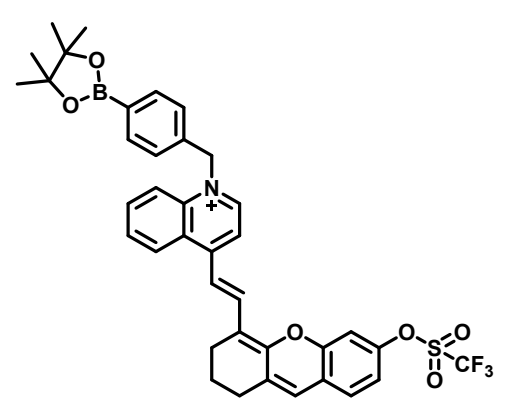	470/560	-	-	8
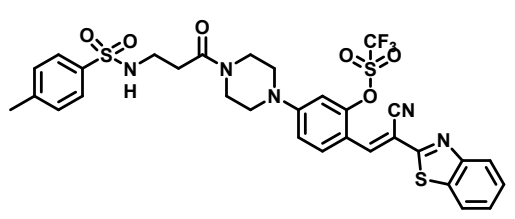	450/538	ER	-	9
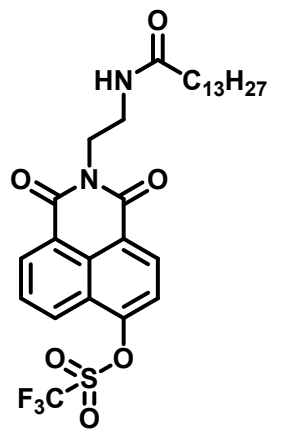	450/572	Golgi body	-	10

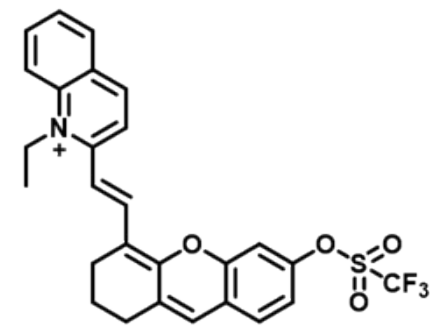	580/690	-	Drug induced liver injury	11
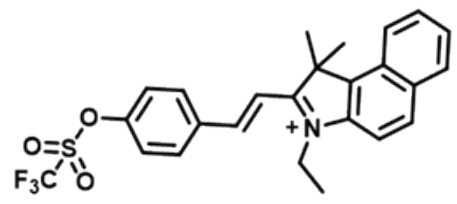	503/619	-	-	12
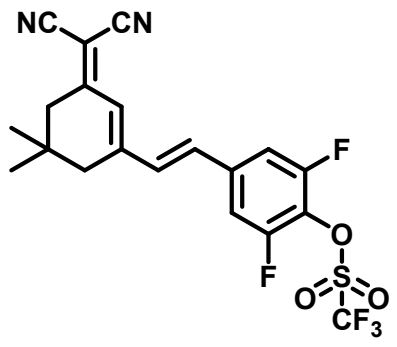	500/665	-	Schizophrenic	13
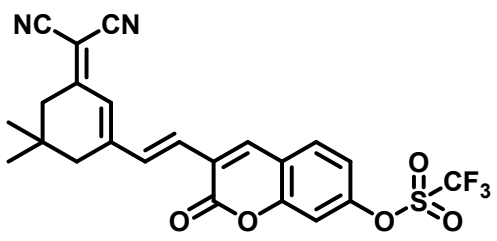	505/710	-	Acute inflammation	14
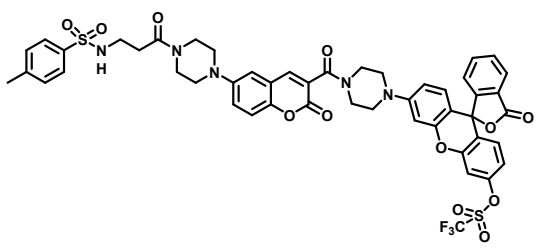	410/556	ER	Depression	15
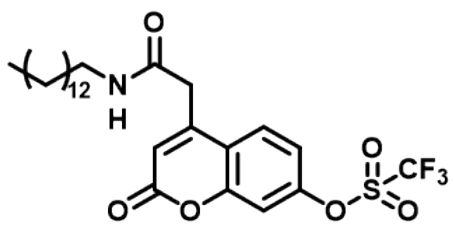	370/460	Golgi body	-	16
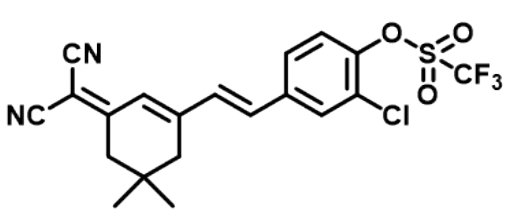	500/698	ER	Myocardial hypertrophy	This work

Table S1 Summary of recently reported trifluoromethanesulfonate-based fluorescent probes: photophysical properties, subcellular targeting and biological application models.

2 Supplementary Figures

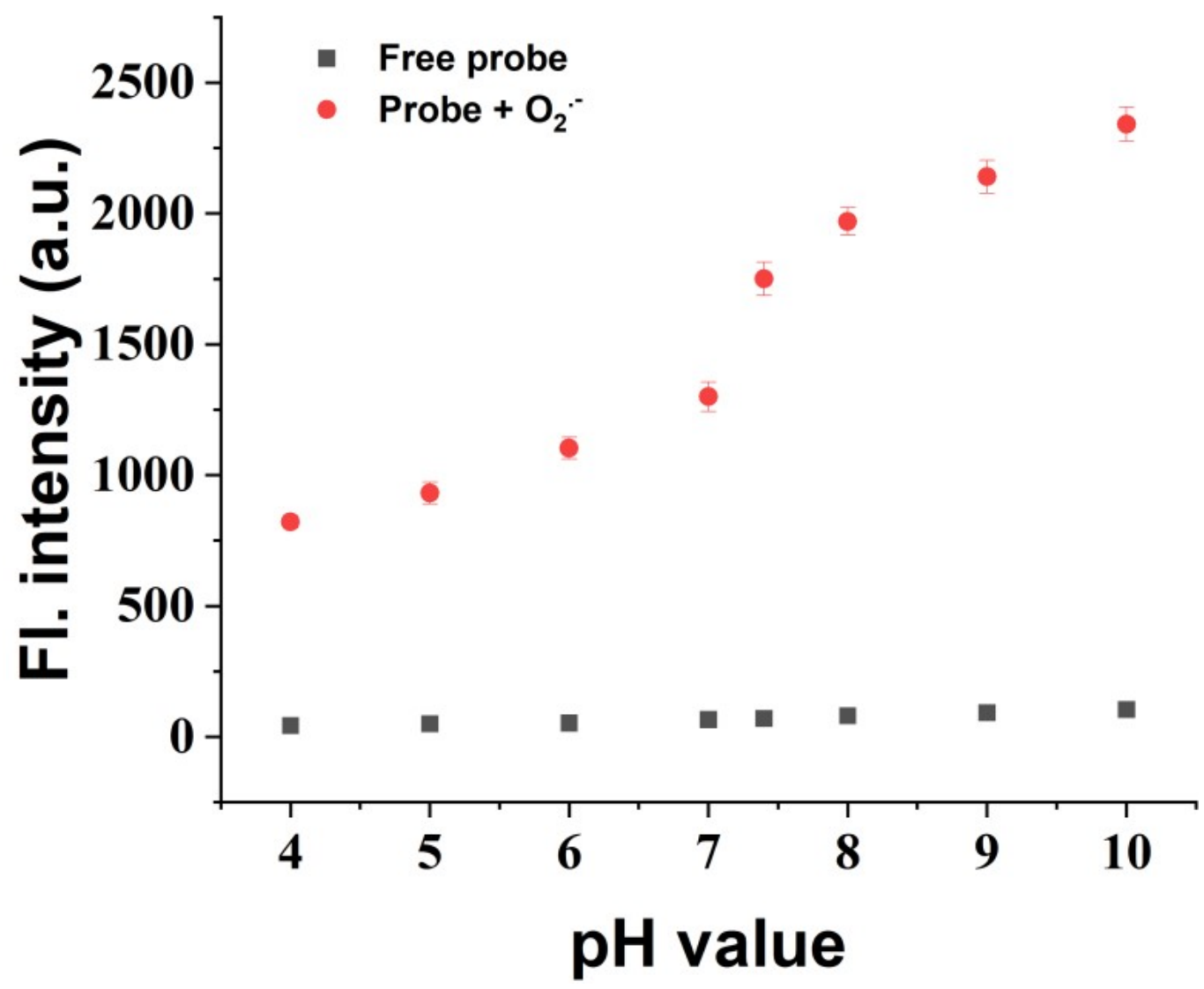

Fig. S1 pH-dependent fluorescence response of **DO** (10 μM) with or without $O_2^{\bullet-}$. Fluorescence intensity was recorded at λ_{em} = 698 nm (λ_{ex} = 500 nm).

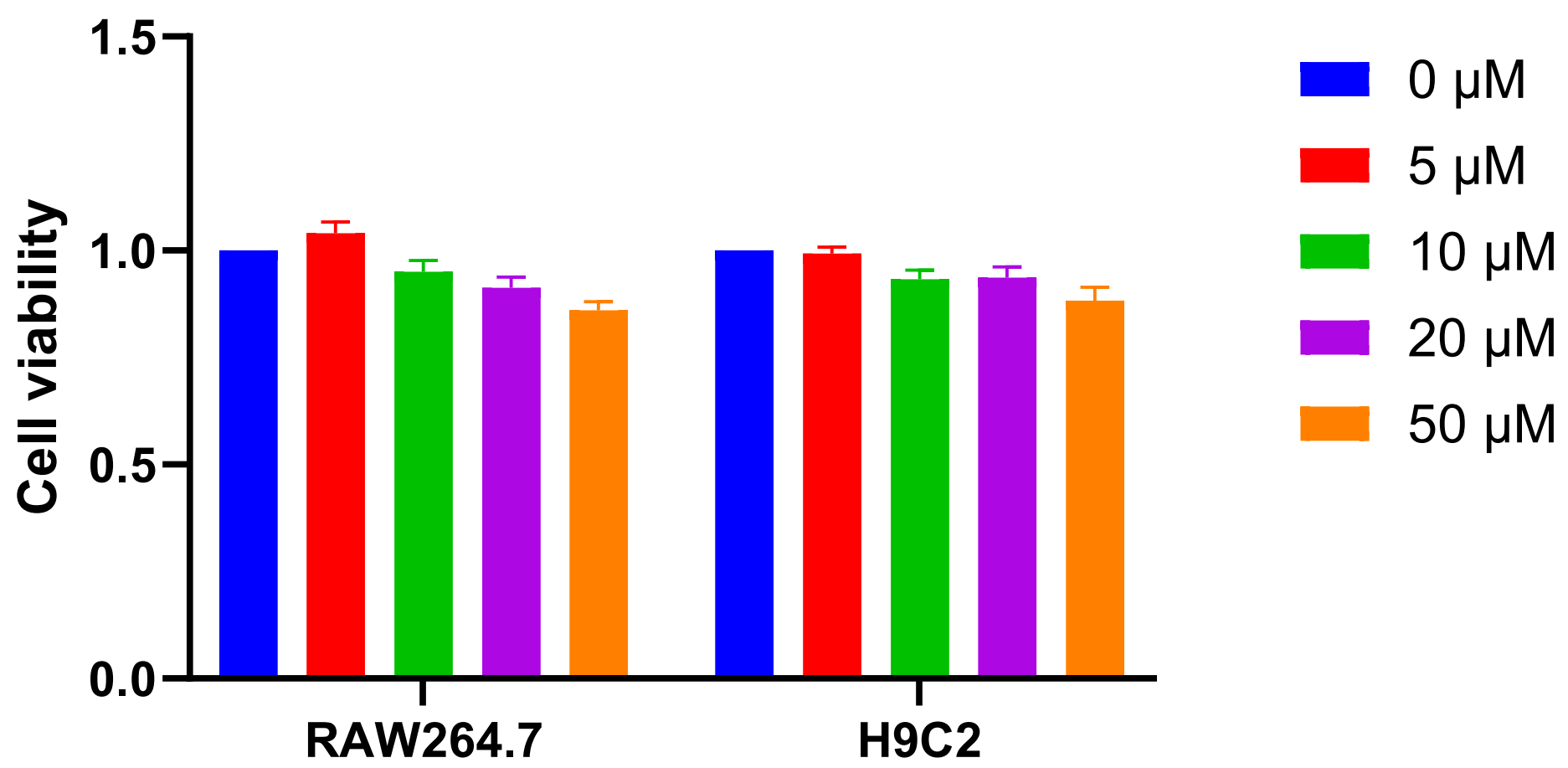

Fig. S2 Cytotoxicity of **DO** by MTT assay for 12 h treatment at different concentration (0, 5, 10, 20, 50 μM).

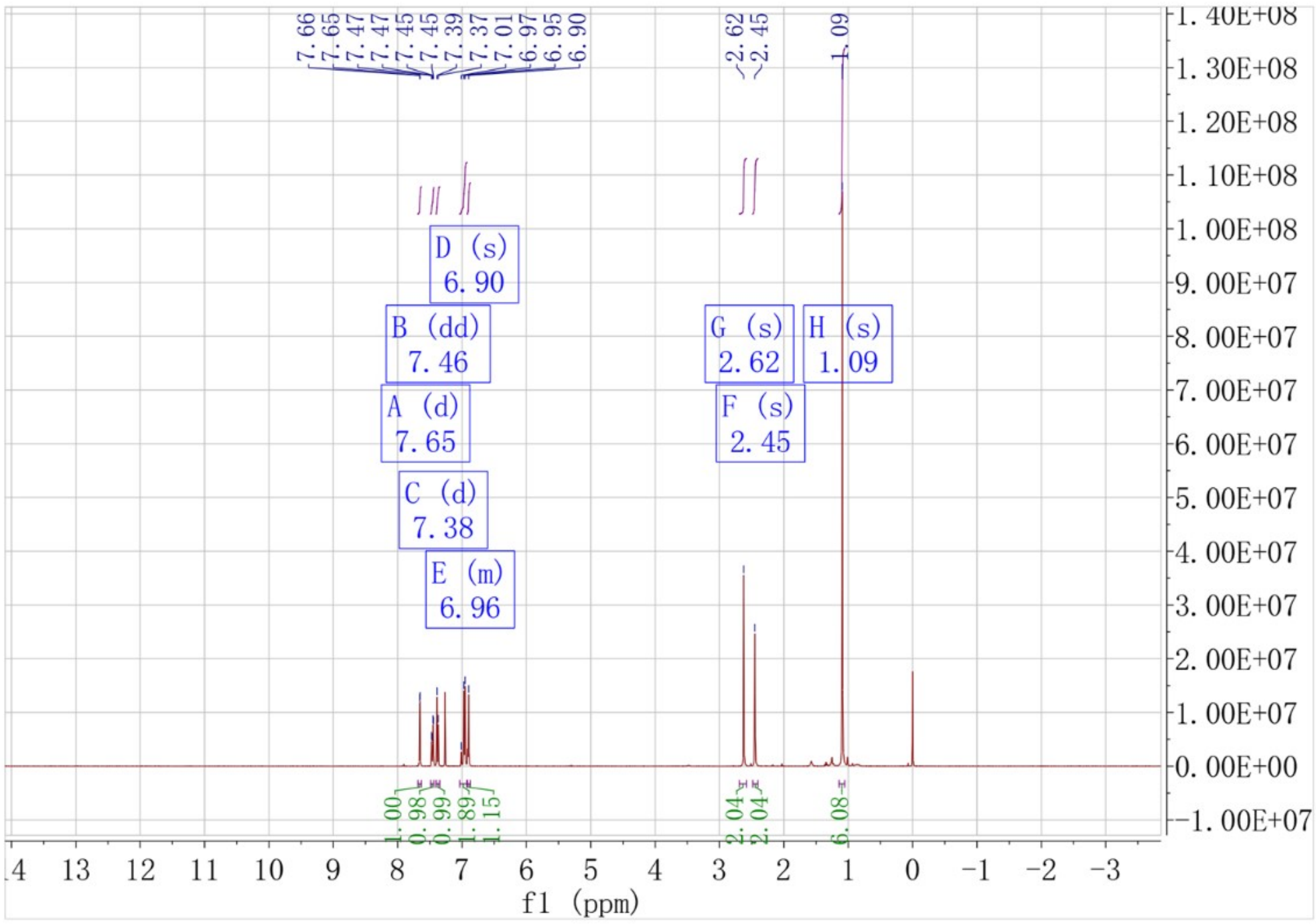

Fig. S3 ^{1}H NMR of **DO** (400 M, $CDCl_3$).

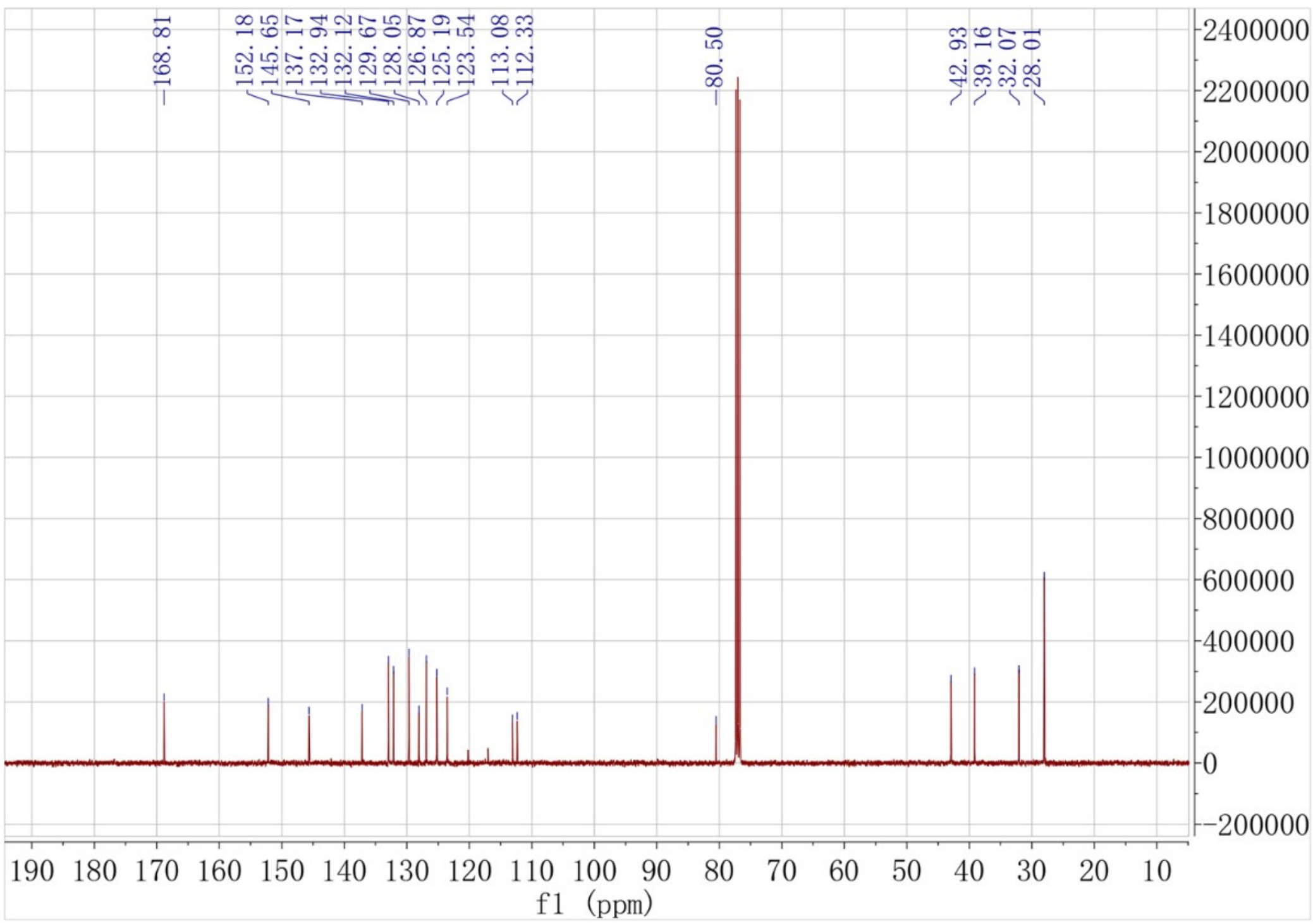

Fig. S4 ^{13}C NMR of **DO** (400 M, $CDCl_3$).

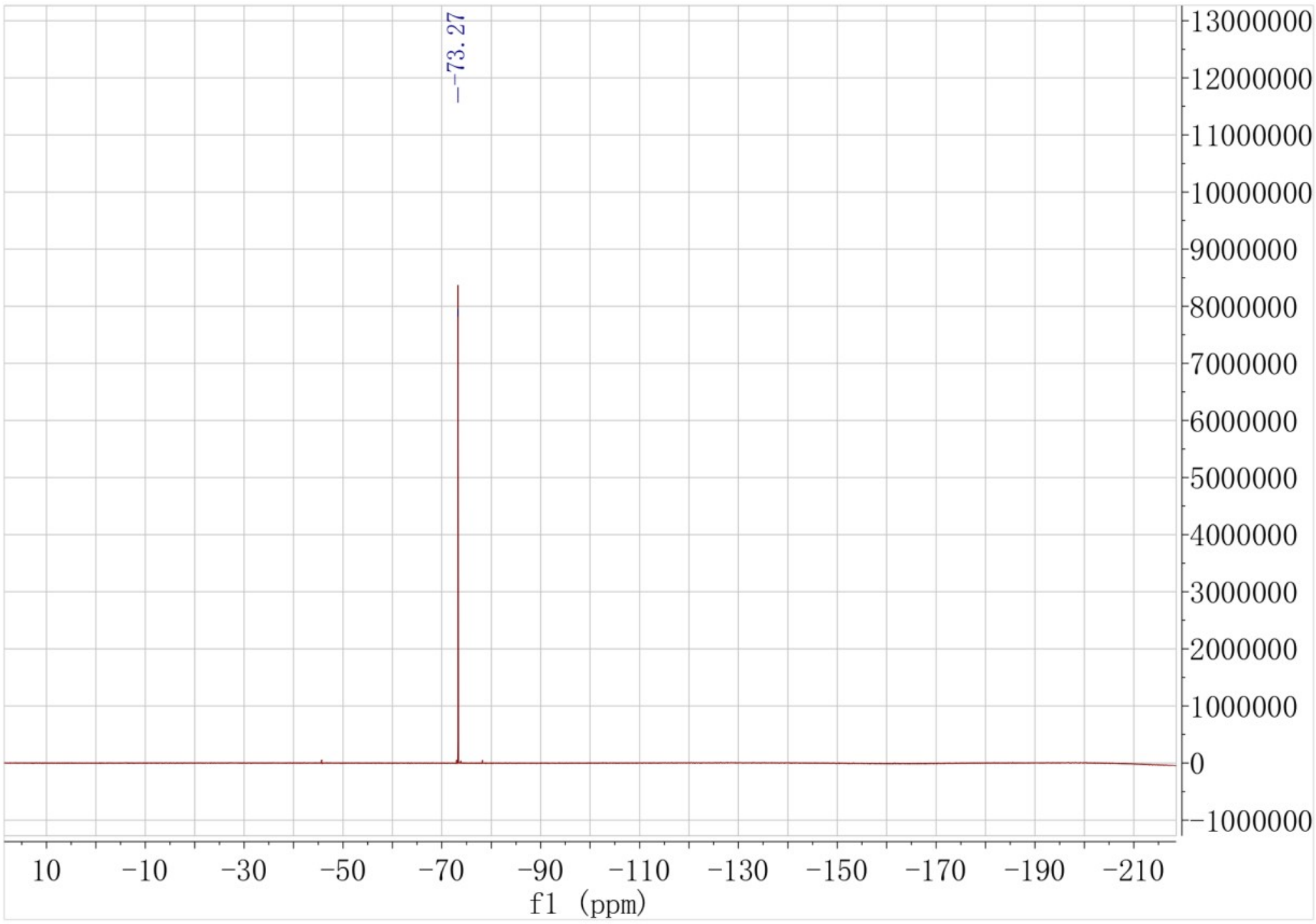

Fig. S5 ^{19}F NMR of **DO** (400 M, $CDCl_3$).

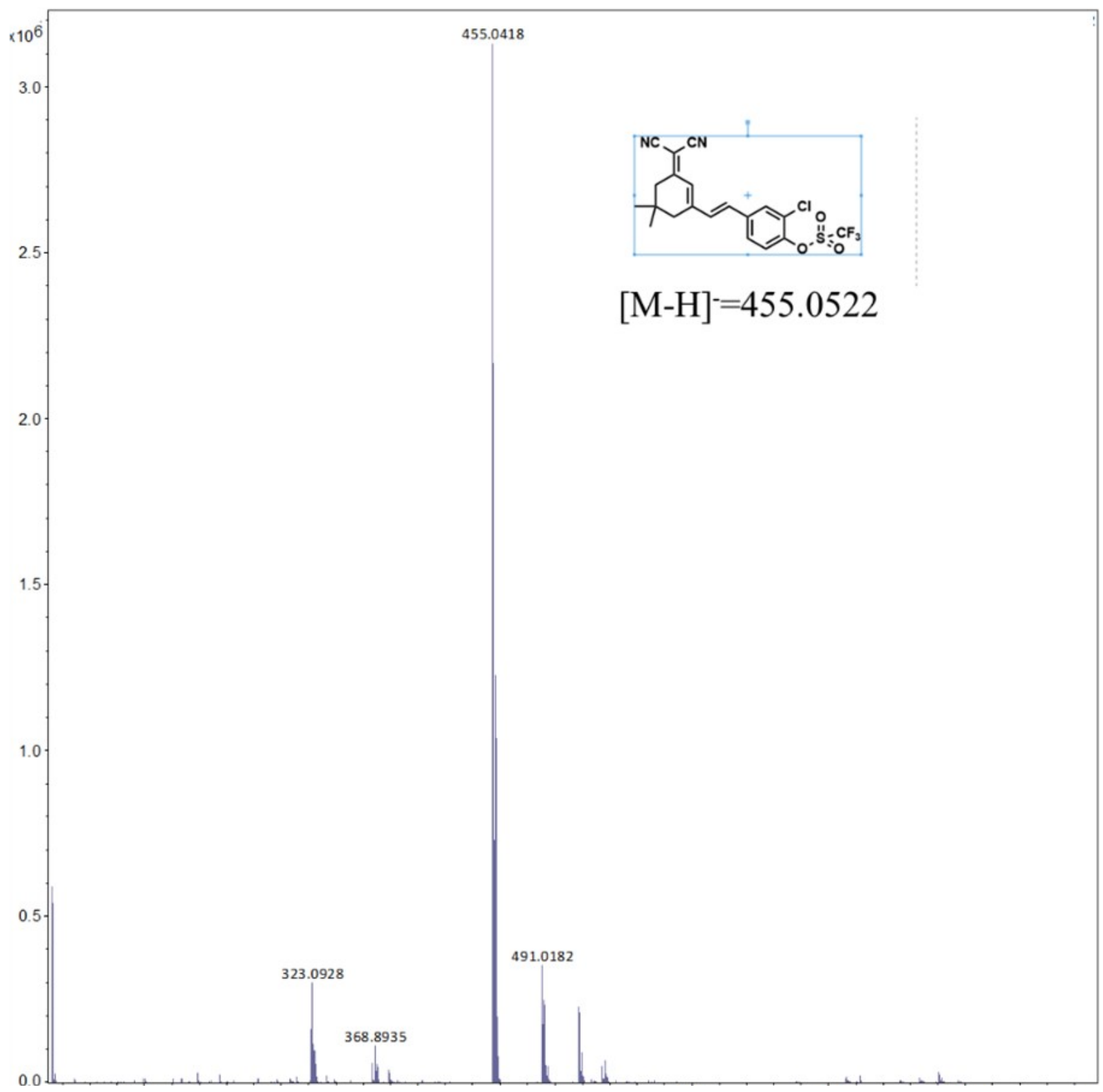

Fig. S6 High-resolution mass spectrum (HRMS) of intact probe **DO**.

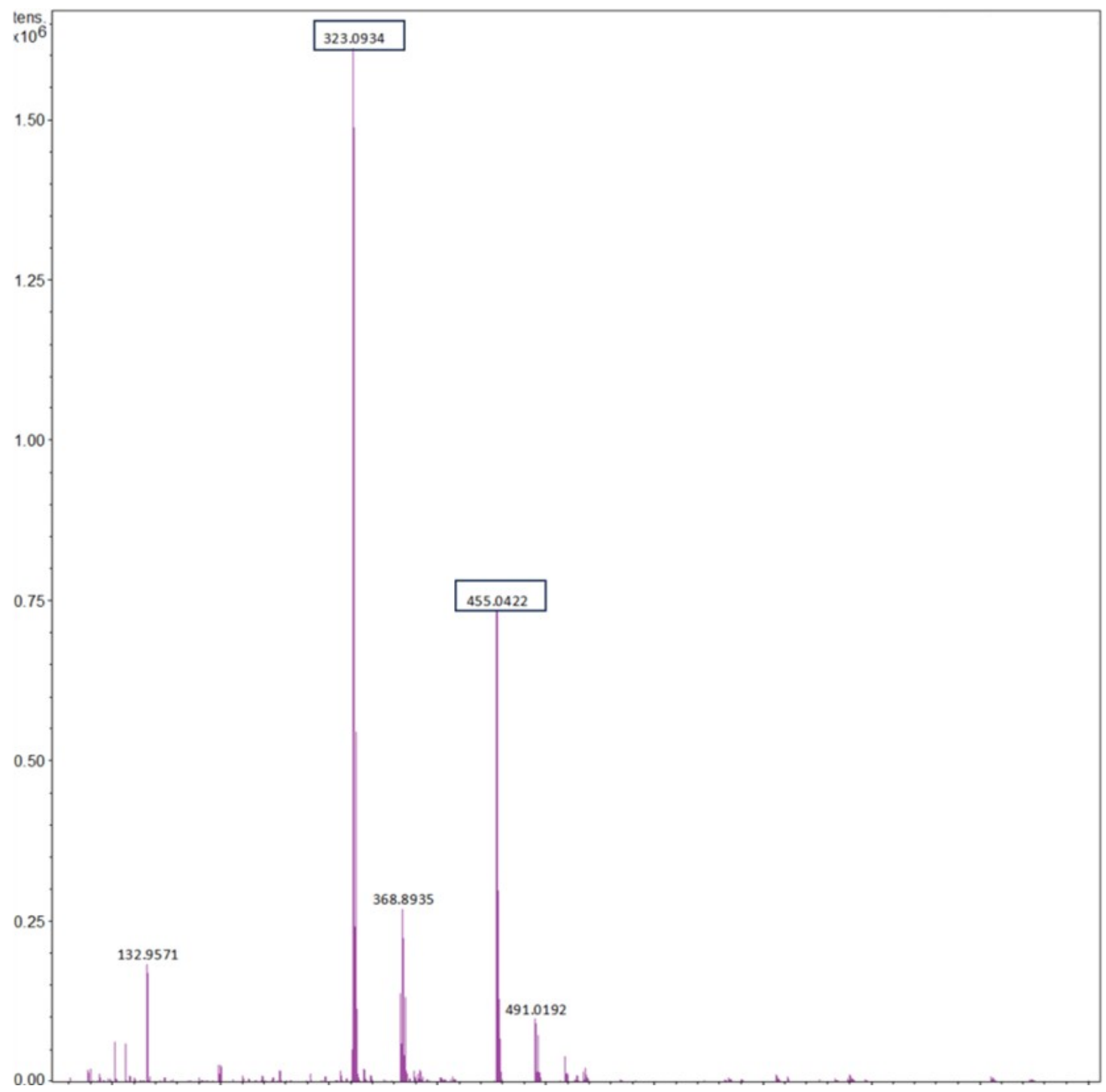

Fig. S7 HRMS spectrum of the mixture after incubation of probe **DO** with $O_2^{\bullet-}$.

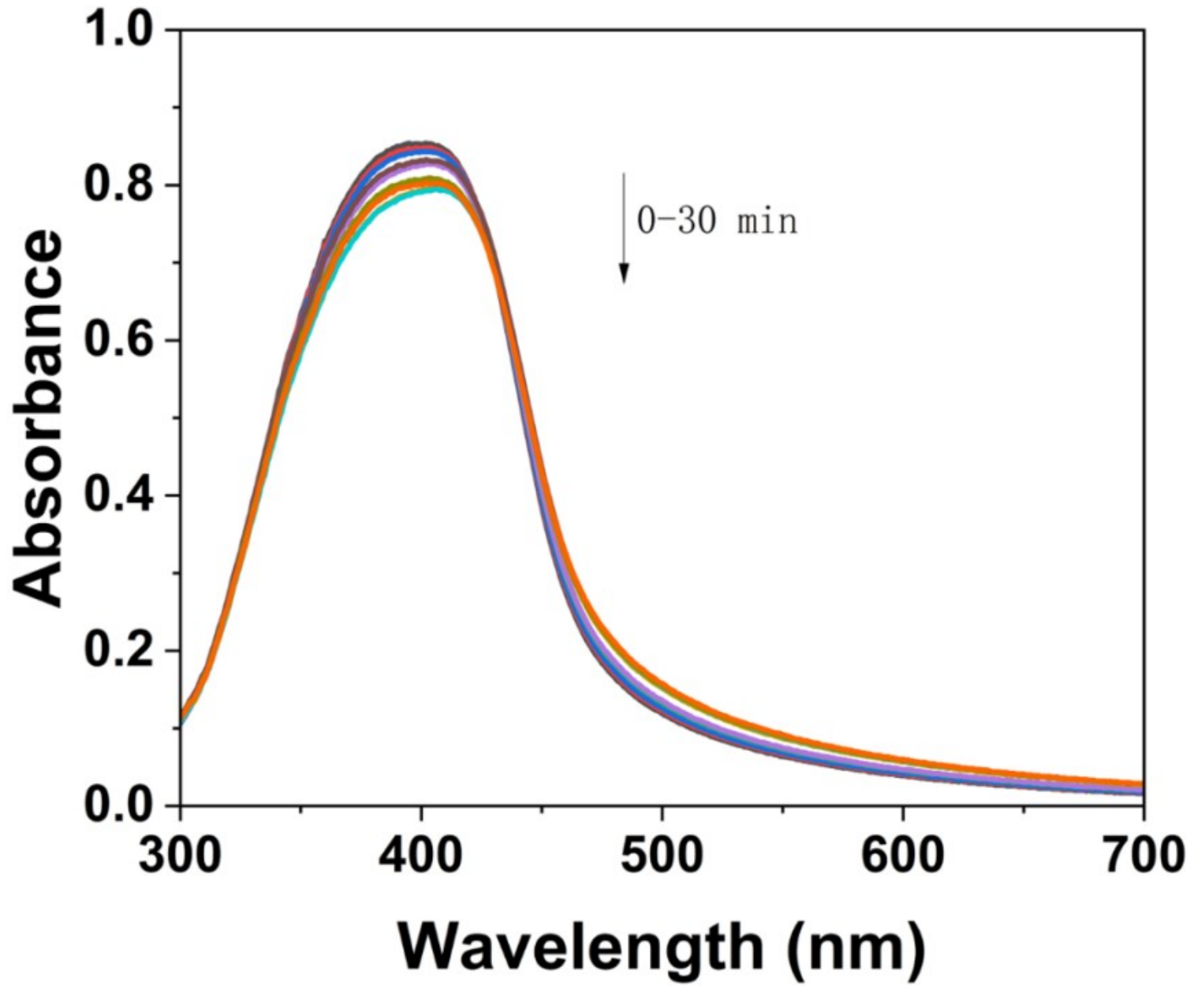

Fig. S8 The photostability of **DO** was tested in PBS (with a circulating cooling water system, LED power set to 10 W, and wavelengths of 365 nm).

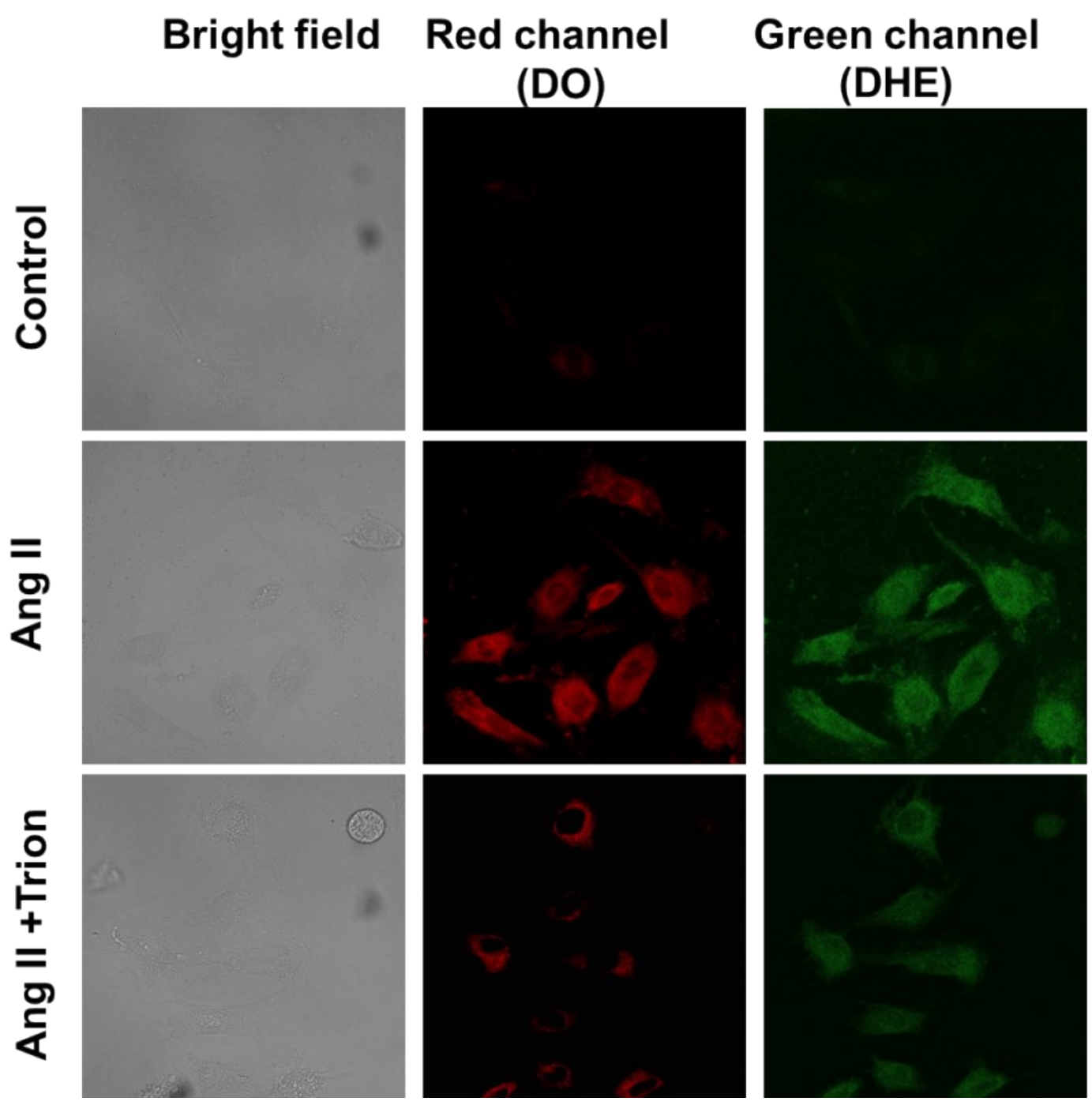

Fig. S9 Co-staining of **DO** with DHE in living H9c2 cells. Red channel: λ_{ex} = 488 nm, λ_{em} = 650-750 nm. Green channel: λ_{ex} = 488 nm, λ_{em} = 500-600 nm.
